# Caspase-8 expression and its Src dependent phosphorylation on Tyrosine 380 triggers NRF2 signaling activation in glioblastoma

**DOI:** 10.1038/s41418-025-01542-3

**Published:** 2025-10-06

**Authors:** Claudia Cirotti, Claudia Di Girolamo, Irene Taddei, Claudia Contadini, Giorgia Massacci, Francesca Sacco, Donatella Del Bufalo, Illari Salvatori, Cristiana Valle, Daniela Barilà

**Affiliations:** 1 Department of Biology, University of Rome “Tor Vergata”, 00133 Rome, Italy; 2 Laboratory of Cell Signaling, IRCCS-Fondazione Santa Lucia, 00179 Rome, Italy; 3 PhD Program in Cellular and Molecular Biology, Department of Biology, University of Rome “Tor Vergata”, 00133 Rome, Italy; 4 Preclinical Models and New Therapeutic Agents Unit, IRCCS Regina Elena National Cancer Institute, 00144 Rome, Italy; 5 Telethon Institute of Genetics and Medicine (TIGEM), Pozzuoli, Italy; 6 Institute of Translational Pharmacology (IFT), Consiglio Nazionale Delle Ricerche (CNR), Rome, Italy; 7 Laboratory of Neurochemestry IRCCS-Fondazione Santa Lucia, 00179 Rome, Italy

**Keywords:** CNS cancer, Oncogenes

## Abstract

Caspase-8 expression is upregulated in many tumors where, despite its canonical apoptotic pathway, it sustains cancer progression promoting cell migration, NF-kB activation and inflammation. Here, we provide the first evidence for a novel role of Caspase-8 in promoting the metabolic rewiring of cancer cells. By performing transcriptomic, proteomic and phosphoproteomic analyses on glioblastoma cellular models, we identify Caspase-8 as an unexpected modulator of NRF2. Here we show that Caspase-8 expression and phosphorylation affect NRF2 activity and mitochondrial homeostasis. Mechanistically, we demonstrate that Src-dependent phosphorylation of Caspase-8 on Tyrosine 380 (Y380), frequently reported in cancers including glioblastoma, sustains mTORC1 activation, thus promoting energy metabolism. mTORC1 activity results in p62 phosphorylation allowing its dependent sequestration of KEAP1 protein and constitutive NRF2 signaling activation, as a consequence. Overall, this work depicted a novel unexpected role for Caspase-8 in the modulation of cancer cell metabolism, bridging together Src, mTORC1 and NRF2 signaling.

## Introduction

Caspase-8 is a cysteine protease that plays a well-known canonical apoptotic function [1]. Consistently, in many tumors the downregulation of its expression contributes to the evasion of apoptosis, one of the hallmarks of cancer cells [1]. Interestingly, some tumors retain high levels of Caspase-8, thereby suggesting that in some specific tumorigenic contexts oncogenic signalings may hijack Caspase-8 function and therefore Caspase-8 expression may turn to be beneficial for cancer cells [2]. Indeed, de novo Caspase-8 expression has been reported in glioblastoma (GBM), compared to normal tissue, and its overexpression has been demonstrated to drive tumor aggressiveness and resistance to therapy in different ways [3–5].

The molecular signaling cascades that allow cancer cells to rewire Caspase-8 function and the signaling pathways through which the aberrant expression of Caspase-8 sustains tumor progression, deserve further elucidation. In this regard, we and others demonstrated that the aberrant activity of Src tyrosine kinase, detected in several tumors, is responsible for Caspase-8 phosphorylation on Tyrosine 380 (Y380); this phosphorylation rewires Caspase-8 function dampening its canonical apoptotic activity [6, 7] and promoting its non-canonical pro-angiogenic and pro-inflammatory functions that overall sustain tumor progression and therapy resistance in GBM [3, 6, 8]. In addition, RNA-seq experiments comparing the transcriptomic profile of glioblastoma cells genetically silenced or not for Caspase-8 expression, highlighted a role of Caspase-8 in the modulation of several signaling pathways [8].

Here, we identify an unexplored crosstalk between Caspase-8 and NRF2 transcription factor, finding a new role of Caspase-8 as modulator of NRF2 signaling pathway. We demonstrate that Caspase-8 modulates mTOR signaling, therefore affecting p62-KEAP1 interaction driving NRF2-dependent metabolic rewiring. Moreover, we identify Src-dependent phosphorylation of Caspase-8 on Y380 as a key mechanism to promote mTORC1 activity and enhance NRF2 signaling in GBM cells, therefore providing evidence for a new key connecting role of Src kinase in cancer.

## Results

### Caspase-8 sustains NRF2 expression and activity in GBM

**Fig. 1:**
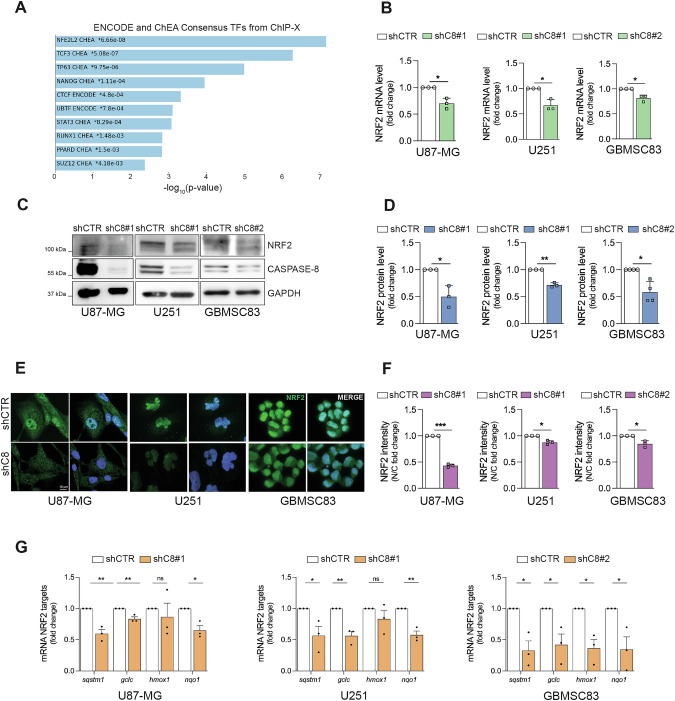
NRF2 expression and activity are strongly downregulated in different glioblastoma cellular models silenced for Caspase-8 expression. **A** Bar chart of top enriched downregulated terms derived from the ENCODE_and_ChEA_Consensus_TFs_from_ChIP-X gene set library by using EnrichR Tool. The top 10 enriched terms for the input gene set (GSE193495) are displayed based on the -log10(p-value), with the actual p-value shown next to each term. The term at the top, NFE2L2, has the most significant overlap with the input query gene set. **B** Real time-PCR of NRF2 gene in U87-MG, U251 and GBMSC83 shCTR and stably silenced for Caspase-8 expression (U87-MG and U251 using shC8#1 sequence, GBMSC83 using shC8#2 sequence) (actin: housekeeping gene). **C** Immunoblotting of NRF2 and Caspase-8 in U87-MG, U251 and GBMSC83 shCTR and stably silenced for Caspase-8 expression (shC8#1 or shC8#2). GAPDH was used as loading control. **D** Densitometric analysis of NRF2 in (**C**) normalized on GAPDH. **E** Representative immunofluorescence of U87-MG, U251 and GBMSC83 shCTR and shC8 cells. NRF2 (green) and DNA (Hoechst, blue) and **F** relative histograms of the NRF2 staining reported as the ratio between nuclear and cytosolic fluorescence intensity (N/C). **G** Real time-PCR of NRF2 target genes (*sqstm1, gclc, hmox1, nqo1*) in U87-MG, U251 and GBMSC83 shCTR and shC8 cells (actin: housekeeping gene). Results represent the mean of at least three independent experiments ( ± SEM). Statistical analyses: paired (**B**, **D**, **F**), and multiple (**G**) *t* test (*P < 0.05; **P < 0.01; ***P < 0.001).

By analyzing our previous transcriptomic data performed on U87-MG cells silenced (shC8#1) or not (shCTR) for *CASP8* [8], we observed a significant downregulation of *NFE2L2* gene expression (Supplementary Fig. S1A) and of NRF2 targets upon the reduction of Caspase-8 levels (Fig. 1A). Interestingly, by using Gepia2 web server [9] we compared the expression levels of *CASP8* and *NFE2L2* genes in tumors and normal tissues from The Cancer Genome Atlas (TCGA) database; the expression of both genes is aberrantly upregulated in several cancers, including GBM (Supplementary Fig. S1B). In addition, we could show a significant positive correlation between *CASP8* and *NFE2L2* expression levels in GBM and other tumor types characterized by high Caspase-8 levels [10] (Supplementary Fig. S1C), supporting the hypothesis of an interplay between Caspase-8 and NRF2 in cancer.

To confirm that Caspase-8 may sustain *NFE2L2* gene expression, NRF2 mRNA and protein levels were evaluated in GBM cell lines and in patient derived cancer stem cells grown as neurospheres stably genetically silenced (shC8) or not (shCTR) for Caspase-8 expression (Fig. 1B–G) [8], as well as in U87-MG cells transiently silenced (siC8) or not (siSCR) for Caspase-8 expression (Supplementary Fig. S1D). Indeed, the expression of NRF2 is significantly decreased in U87-MG, U251 and GBMSC83 shC8 cells, compared to shCTR cells (Fig. 1B–D) and in U87-MG siC8 compared to siSCR cells (Supplementary Fig. S1D). Immunofluorescence analyses (Fig. 1E, F and Supplementary Fig. S1E, F) and subcellular fractionation (Supplementary Fig. S1G) further confirmed that Caspase-8 expression promotes NRF2 nuclear localization. Consistently, Caspase-8 expression sustains NRF2 transcriptional activity as demonstrated by evaluating the expression levels of some NRF2 target genes assayed by real-time qPCR experiments (Fig. 1G).

### Caspase-8 expression modulates the proteomic and phospho-proteomic profile impinging on mTOR signaling

To further investigate the role of Caspase-8 expression in the modulation of GBM cell signaling, we performed a deep mass spectrometry (MS)-based (phospho)proteomic analysis of U87-MG cells silenced (shC8#1) or not (shCTR) for Caspase-8 expression. By using a label-free quantification approach coupled with a data-independent acquisition (DIA) method, we were able to quantify about 6,500 proteins (Table S1/ Dataset 1) and 12,189 phosphosites mapping to 2696 protein groups (Table S2/Dataset 2) (Supplementary Fig. S2A, B).

**Fig. 2:**
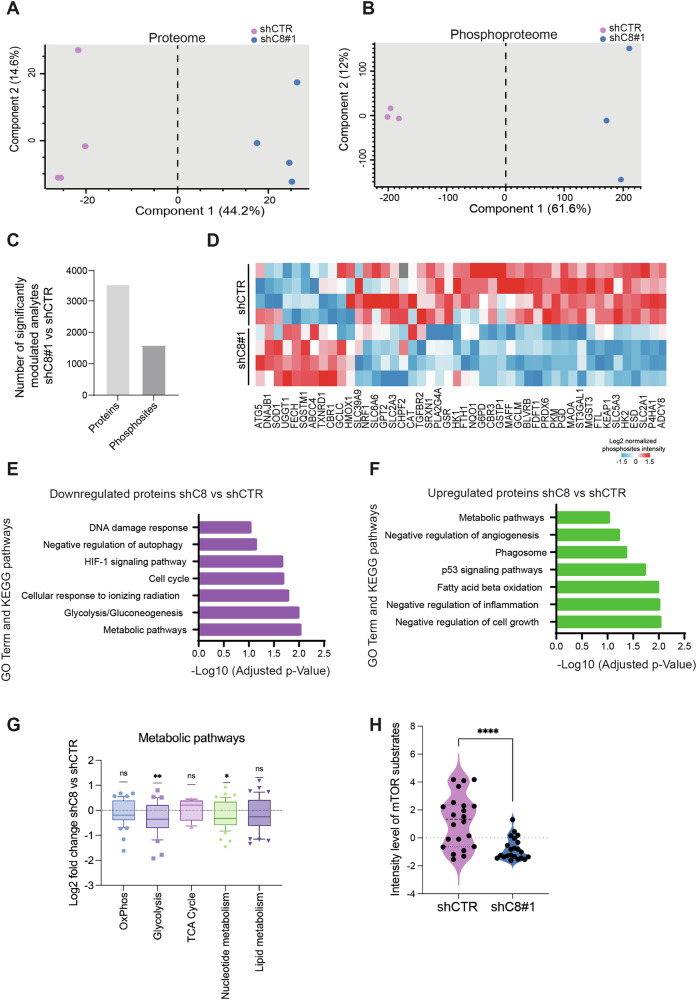
Proteomic analysis reveals a crosstalk between Caspase-8 and NRF2. **A**, **B** Principal Component Analysis (PCA) of the analytes quantified across proteome (**A**) and phosphoproteome (**B**) replicates. **C** Barplot reporting the number of significantly modulated proteins and phosphosites in shC8 compared to shCTR cells. **D** Heatmap of protein abundance of the NRF2 targets quantified. **E**, **F** Bar plots reporting the GO-biological processes and KEGG pathways enriched by the proteins that are significantly downregulated (**E**) or upregulated (**F**) in shC8 compared to shCTR cells. **G** Box plot of modulated proteins involved in specific metabolic pathways reported as the median log2 fold change in shC8 cells vs shCTR cells. **H** The phosphorylation intensity was normalized on the total median intensity of all the quantified phosphosites in each experimental condition. Then the normalized intensity of the significantly modulated mTOR substrates between shC8 and shCTR cells was reported in the violin plot.

Notably, our strategy showed to be efficient as we were able to measure the total protein abundance for 89% of the phosphosites (Supplementary Fig. S2C). Additionally, the biological replicates showed strong correlation, with Pearson correlation coefficients ranging from 0.6 to 0.9 (Supplementary Fig. S2D, E). First, we examined whether our proteomic and phosphoproteomic profiling could distinguish between shC8 and shCTR samples. As shown in the Principal Component Analysis (PCA), component 1 clearly segregated shCTR from shC8 cells at both the proteome and phosphoproteome levels (Fig. 2A, B). Student’s T Test analysis of our data revealed that this segregation is due to the statistically significant modulation of ~3500 proteins and ~1600 phospho-sites (Fig. 2C), including Caspase-8 protein expression (Supplementary Fig. S2F). In agreement with the transcriptome dataset, we also observed a trending downregulation of NRF2-target proteins after Caspase-8 silencing (Fig. 2D). Next, we investigated Caspase-8 silencing effects on crucial biological processes. We then conducted gene ontology term enrichment analysis on our proteomic data. Our findings indicate that Caspase-8 silencing significantly affects pathways related to metabolism, autophagy, cell cycle and DNA damage response (Fig. 2E, F). Of note, by focusing on metabolic pathways that appears to be mostly affected by Caspase-8 expression (Fig. 2E, F), we observed that among modulated proteins, those involved in glycolysis and nucleotide metabolism are significantly downregulated, together with a trending downregulation of protein involved in oxidative phosphorylation and lipid metabolism and a slight increase in TCA cycle proteins (Fig. 2G, Table S4).

Being mTORC1 kinase one of the main players in the regulation of cell growth and metabolism, frequently aberrantly activated in cancer [11], we asked for a role of mTORC1 in our experimental models. To this aim, we compared the proteome and phosphoproteome changes and we found that, when normalizing for the protein level, more than 50% of the phosphosites still remained significantly modulated and only 13% of the proteins are modulated both at the phosphorylation and total protein levels (Supplementary Fig. S2G). These data suggest that Caspase-8 expression profoundly impacts cell signaling also at a post-translational level. Interestingly, among the significantly modulated phosphosites our analysis revealed that canonical mTOR substrates were significantly hypophosphorylated in shC8 cells compared to shCTR cells (Fig. 2H, Table S3), suggesting that the expression of Caspase-8 strongly sustains the activation of mTOR dependent signaling.

### Caspase-8 expression promotes p62-KEAP1 interaction via mTORC1 modulation

It has been previously shown that p62-KEAP1 interaction relies on mTORC1-dependent p62 phosphorylation on Serine 349 (p-p62^S349^) [12]. Interestingly, data from literature recently reported that in GBM cells high expression levels of NRF2 protein are sustained by this mechanism [13].

**Fig. 3:**
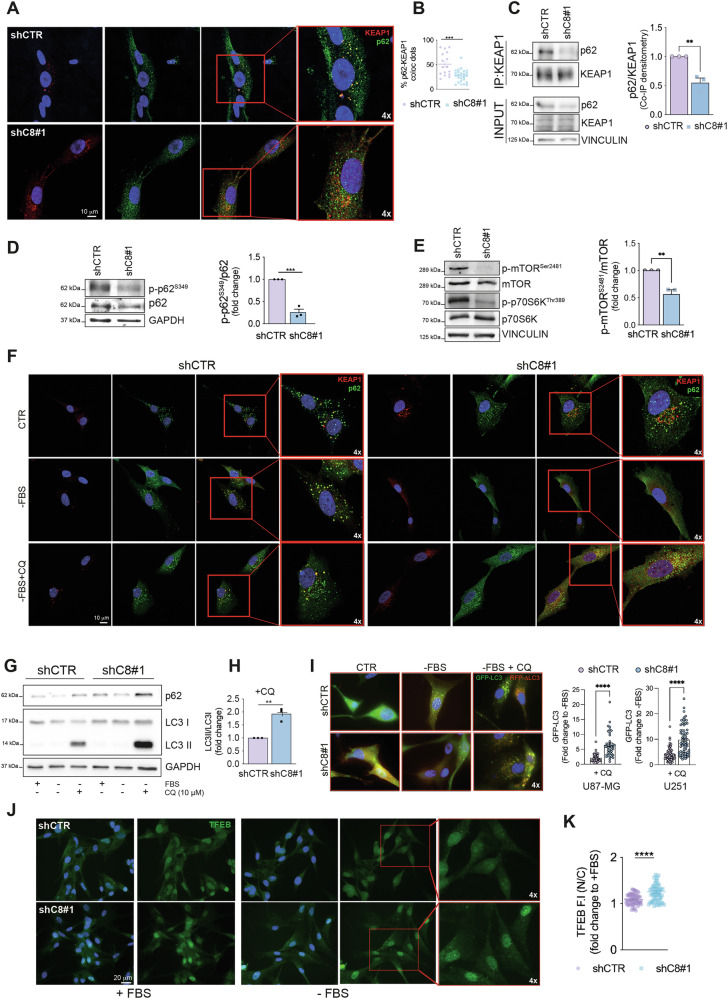
Caspase-8 overexpression sustains p62-KEAP1 interaction, via mTORC1 activation. **A** Confocal microscopy analyses and **B** quantification of colocalizing dots in U87shCTR and shC8#1 cells; p62 (green), KEAP1 (red), DNA (Hoechst, blue); 4× digital magnification showing merged signals. **C** Co-immunoprecipitation and immunoblotting experiments (left) and relative quantification (right) of KEAP1 and p62 proteins in U87shCTR and shC8#1 cells; **D** Immunoblotting of p-p62^S349^ and p62 in U87shCTR and U87shC8 cells and relative densitometric analysis of p-p62^S349^ normalized on total p62. GAPDH was used as loading control. **E** Immunoblotting of p-mTOR^Ser2481^, mTOR, p-p70S6K^Thr389^ and p70S6K in U87shCTR and shC8 cells and relative densitometric analysis of p-mTOR^Ser2481^ normalized on total mTOR. Vinculin was used as loading control. **F** Confocal microscopy analyses in U87shCTR and U87shC8 cells upon serum deprivation (-FBS) for 2 hours and treated with CQ 10 μM for 16 hours; p62 (green), KEAP1 (red), DNA (Hoechst, blue); 4x digital magnification showing merged signals. **G** Immunoblotting of p62, LC3 I and II in U87shCTR and U87shC8 cells upon serum deprivation (-FBS) for 2 hours and chloroquine (CQ) 10 μM for 16 hours. GAPDH was used as loading control. **H** Histogram representing the densitometric analysis of LC3 II normalized on LC3 I reported as the fold change between U87shCTR and shC8#1 cells upon chloroquine (CQ) treatment, as in (**G**). **I** Representative immunofluorescence (ApoTome) in U87-MG (left) and quantification (right) of GFP-LC3 signal in U87shCTR and U87shC8#1 cells stably expressing GFP-LC3-RFP-LC3ΔG probe, upon serum deprivation (-FBS) for 2 hours and chloroquine (CQ) 10 μM for 16 hours. The quantification of the same experiment carried out in U251shCTR and U251shC8#1 is also shown. GFP-LC3 (green), RFP-ΔLC3 (red), DNA (Hoechst, blue); 4x digital magnification showing merged signals. **J** Immunofluorescence of U87shCTR and U87shC8 cells upon serum deprivation (-FBS); TFEB (green), DNA (Hoechst, blue); 4× digital magnification showing merged signals. **K** Quantification of TFEB staining reported as the ratio between nuclear and cytosolic fluorescence intensity (N/C) in U87shCTR and U87shC8#1 cells. Results represent the mean of at least three independent experiments ( ± SEM). Statistical analyses: paired (**C**, **D**, **E**, **H**) and unpaired (**B**, **I**, **K**) *t* test (**P < 0.01; ***P < 0.001; ****P < 0.0001).

We therefore asked the question whether Caspase-8 may modulate NRF2 expression by impinging on this pathway. As expected, confocal microscopy analysis showed a strong colocalization between p62 and KEAP1 in shCTR cells. On the contrary, silencing of Caspase-8 expression significantly prevented this interaction (Fig. 3A, B and Supplementary Fig. S3A, B), supporting our hypothesis. In addition, co-immunoprecipitation experiments confirmed p62-KEAP1 interaction (Fig. 3C). Interestingly, we could show that p-p62^S349^ levels were significantly reduced upon silencing of Caspase-8 expression compared to control cells (Fig. 3D and Supplementary Fig. S3C). Consistently, by analyzing the phosphorylation of mTOR on Serine 2481 (p-mTOR^Ser2481^) and of its well-known target p70S6 kinase on threonine 389 (p-p70S6K^Thr389^), we could confirm that the downregulation of Caspase-8 expression drives a significant reduction of mTORC1 signaling (Fig. 3E and Supplementary Fig. S3D). These data confirm our proteomic and phosphoproteomic studies and highlight a role for Caspase-8 in the modulation of mTORC1-dependent pathways in glioblastoma.

mTORC1 activation in cancer sustains anabolism and cancer cell growth [14] and it is intriguingly connected to p62 and NRF2 pathway activation [15]. Being p62 a key adaptor protein in autophagy machinery, we asked whether Caspase-8 expression may affect autophagic flux causing p62 accumulation in glioblastoma. Of note, confocal microscopy analysis demonstrated that autophagy induction by serum deprivation caused the reduction of p62 positive bodies in shC8 cells thus preventing its interaction with KEAP1 (Fig. 3F and Supplementary Fig. S3E). Moreover, autophagy inhibition by chloroquine (CQ) treatment rescued p62-KEAP1 bodies in shC8 cells. Conversely, no differences were observed in shCTR cells, suggesting a role for Caspase-8 in impairing both p62-KEAP1 interaction and autophagy (Fig. 3F and Supplementary Fig. S3E).

Accordingly, immunoblotting of autophagic markers p62 and LC3 showed an increase in autophagic flux upon serum deprivation in shC8 cells (Fig. 3G, H and Supplementary Fig S3F, G), where LC3-I conversion to LC3-II, reported as fold change of their ratio in chloroquine-treated samples, is significantly higher compared to shCTR cells (Fig. 3H).

To strengthen our data, we also took advantage of the autophagic flux probe GFP-LC3-RFP-LC3ΔG, composed by RFP-LC3ΔG that cannot undergo lipidation and thus used as internal control, and GFP-LC3 that is degraded after fusion with lysosomes [16]. By immunofluorescence experiments, we demonstrated that the expression of Caspase-8 slows down autophagic flux as no significant decreased in GFP-LC3 was observed upon serum deprivation in shCTR cells (Fig. 3I and Supplementary Fig. S3H). On the contrary, shC8 cells showed a decrease in GFP-LC3 by immunofluorescence; no modulation in RFP-LC3ΔG was coherently detected in both cell lines (Fig. 3I and Supplementary Fig. S3H). These data reinforce the inhibitory role of Caspase-8 on autophagy, in line with our proteomic analyses identifying negative regulation of autophagy among pathways downregulated by Caspase-8 silencing (Fig. 2E).

Interestingly, our proteomic data showed a significant downregulation of mLST8 (Supplementary Fig. S3I), key component of mTORC1 complex and responsible for its sustained activation [14]. In addition, a significant modulation of HDAC6 was observed (Supplementary Fig. S3J), the latter responsible for the modulation of autophagy-related transcription factors, namely FOXO1 and TFEB [17, 18].

Accordingly, we investigated the subcellular localization of the Transcription Factor EB (TFEB), a member of the bHLH leucine-zipper family of transcription factors, master regulator of clearance pathways and a direct mTORC1 target [19]. mTORC1 promotes TFEB phosphorylation on Serine 211 causing the retention of the transcription factor in the cytosol and preventing the expression of multiple genes implicated in autophagy and lysosomal function [20]. Here we show that silencing of Caspase-8 expression increased TFEB nuclear localization, which is further enhanced upon autophagy stimulation through serum deprivation compared to control cells (Fig. 3J, K Supplementary Fig. S3K, L), without affecting TFEB overall levels (Supplementary Fig. S3M).

Overall, these data confirm that Caspase-8 expression in GBM cells sustains mTORC1 activity and autophagy inhibition.

### Caspase-8 phosphorylation on Y380 promotes p62-KEAP1 interaction, thus enhancing NRF2 signaling

We have previously shown that Src hyperactivation in GBM drives Caspase-8 phosphorylation on Y380, therefore rewiring Caspase-8 function that in this context sustains angiogenesis and inflammation [3, 7, 8]. Importantly, we also reported that Src kinase promotes NRF2 expression and activity in GBM cells [21]. We therefore asked whether Src may join Caspase-8 to NRF2 signaling.

**Fig. 4:**
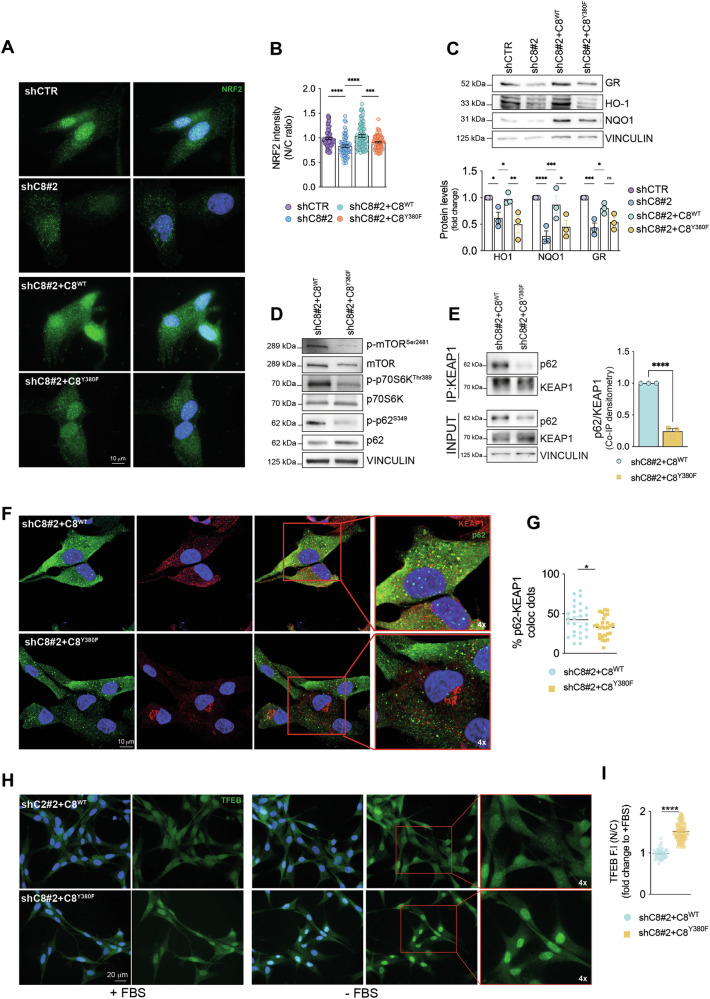
Caspase-8 phosphorylation on Tyrosine 380 sustains NRF2 activation. **A** Immunofluorescence in U87shCTR, U87shC8#2, U87shC8#2 + C8^WT^ and U87shC8#2 + C8^Y380F^ cells: NRF2 (green) and DNA (Hoechst, blue). **B** Quantification of NRF2 staining in A) reported as the ratio between nuclear and cytosolic fluorescence intensity (N/C). **C** Immunoblotting and relative densitometric analyses of GR, HO-1 and NQO1 in U87shCTR, U87shC8#2, U87shC8#2 + C8^WT^ and U87shC8#2 + C8^Y380F^ cells. Vinculin was used as loading control. **D** Immunoblotting of p-mTOR^Ser2481^, mTOR, p-p70S6K^Thr389^, p70S6K, p-p62^S349^ and p62 in U87shC8#2 + C8^WT^ and U87shC8#2 + C8^Y380F^ cells. Vinculin was used as loading control. **E** Co-immunoprecipitation experiments (left) and relative quantification (right) of KEAP1 and p62 proteins in U87shC8#2 + C8^WT^ and U87shC8#2 + C8^Y380F^ cells. **F** Confocal microscopy analyses and **G** quantification of colocalizing dots in U87shC8#2 + C8^WT^ and U87shC8#2 + C8^Y380F^ cells; p62 (green), KEAP1 (red), DNA (Hoechst, blue); 4× digital magnification showing merged signals. **H** Representative immunofluorescence of U87shC8#2 + C8^WT^ and U87shC8#2 + C8^Y380F^ cells upon serum deprivation (-FBS); TFEB (green), DNA (Hoechst, blue); 4× digital magnification showing merged signals. **I** Quantification of TFEB staining of **H** reported as the ratio between nuclear and cytosolic fluorescence intensity (N/C). Results represent the mean of at least three independent experiments ( ± SEM). Statistical analyses: paired (**C**, **E**) and unpaired *t* test (**B**, **G**, **I**) (*P < 0.05; ***P < 0.001; ****P < 0.0001).

To this aim, we tested whether Src-mediated Caspase-8 phosphorylation on Y380 may contribute to NRF2 modulation, taking advantage of U87-MG and U251 shC8 cells reconstituted with either Caspase-8 *wild type* (shC8 + C8^WT^) or Caspase-8 Y380F (shC8 + C8^Y380F^), the non-phosphorylatable mutant [8] (Supplementary Fig. S4A). Importantly, the reconstitution of C8^WT^ expression sustains NRF2 nuclear localization and activity (Fig. 4A–C and Supplementary Fig. S4B–E). On the contrary, the C8^Y380F^ failed to upregulate NRF2, as well as its canonical targets HO-1, NQO1 and GR (Fig. 4A–C and Supplementary Fig. S4B–E). Consistently, C8^WT^ but not C8^Y380F^ supported mTORC1 activation and p62 phosphorylation on S349 (Fig. 4D and Supplementary Fig. S4F) and, therefore, p62-KEAP1 interaction and colocalization (Fig. 4E–G and Supplementary Fig. S4G, H).

Coherently, we observed that shC8 cells reconstituted for C8^WT^ expression significantly showed a lower nuclear accumulation of TFEB transcription factor compared to the shC8 cells reconstituted for C8^Y380F^ expression, both in basal conditions and upon serum deprivation (Fig. 4H, I and Supplementary Fig. S4I, J).

Overall, these data highlight a role for Src-dependent Caspase-8 phosphorylation on Y380 in the mTORC1-dependent constitutive activation of NRF2 in cancer.

### Caspase-8 and its phosphorylation on Y380 ensure energy metabolism affecting mitochondrial homeostasis

**Fig. 5:**
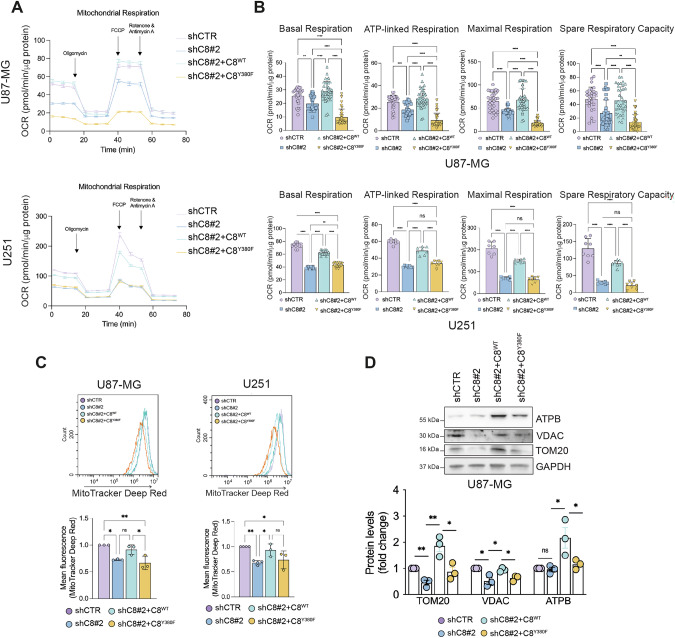
Caspase-8 expression and phosphorylation on Y380 ensures energy metabolism and mitochondrial activity. **A** Representative profile of Seahorse assay showing the oxygen consumption rate (OCR) of U87 and U251 shCTR, shC8#2, shC8#2 + C8^WT^ and shC8#2 + C8^Y380F^ and **B** relative histograms of basal respiration, ATP-linked respiration, maximal respiration and spare respiratory capacity. **C** Cytofluorimetric analyses of active mitochondria upon MitoTracker Deep Red staining and relative quantification in U87 and U251 shCTR, shC8#2, shC8#2 + C8^WT^ and shC8#2 + C8^Y380F^. **D** Immunoblotting of ATPB, VDAC, TOM20 and relative densitometric analyses in U87shCTR, U87shC8#2, U87shC8#2 + C8^WT^ and U87shC8#2 + C8^Y380F^ cells. GAPDH was used as loading control. Results represent the mean of at least three independent experiments ( ± SEM). Statistical analyses: One-way ANOVA test (**B**) and paired *t* test (**C**, **D**) (*P < 0.05; ***P < 0.001; ****P < 0.0001).

To clarify whether Caspase-8 expression and phosphorylation on Y380 may modulate glioblastoma cells metabolism, we performed bioenergetic analysis through Seahorse Bioscience technology. We demonstrated that shC8 cells exhibited mitochondrial impairment with a reduction in basal respiration, ATP-linked respiration, and maximal respiration, along with the spare respiratory capacity (Fig. 5A, B). The latter two parameters indicating the cellular capacity to respond to increased energy demand. Notably, the reconstitution of U87shC8 and U251shC8 cells with C8^WT^, but not with C8^Y380F^, was sufficient to restore the observed phenotype (Fig. 5A, B).

Cytofluorimetric analyses of mitochondrial mass by using MitoTracker Green probe suggest that Caspase-8 silencing does not decrease mitochondrial content, with the only exception for a slight decrease observed in C8^Y380F^ overexpressing cells (Supplementary Fig. S5A), in line with no significant modulation of mitochondrially encoded genes, *MT-ND2* (ND2), *MT-ND5* (ND5) and *MT-CO1* (COX1) (Supplementary Fig. S5B).

On the contrary, cytofluorimetric analyses of mitochondrial activity by using MitoTracker Deep Red probe that selectively stains active mitochondria, show lower mitochondrial activity in shC8 and shC8 + C8^Y380F^ cells compared to shCTR and shC8 + C8^WT^ cells. This data indicates that despite no difference in mitochondrial content Caspase-8 expression affects mitochondrial functionality (Fig. 5C).

Of note, immunofluorescence and immunoblotting experiments revealed a decrease in the expression of the mitochondrial marker TOM20 in shC8 cells, which was fully rescued by the expression of C8^WT^ and only partially by C8^Y380F^ (Supplementary Fig. S5C and Fig. 5D). Importantly, the evaluation of the levels of ATP synthase β subunit (ATPB) and Voltage-dependent anion channel (VDAC), mitochondrial proteins, both overexpressed in glioblastoma and linked to tumor growth and poor prognosis [22, 23], show their significant modulation among the experimental conditions (Fig. 5D). These data reinforce the role of *wild-type* Caspase-8 in metabolic rewiring, allowing the upregulation of mitochondrial proteins.

Taken together these results suggested that Caspase-8 expression and its phosphorylation on Y380 sustains GBM energy demand, affecting mitochondrial homeostasis.

### Reconstitution of NRF2 expression rescues the mitochondrial functionality of GBM shC8 cells

**Fig. 6:**
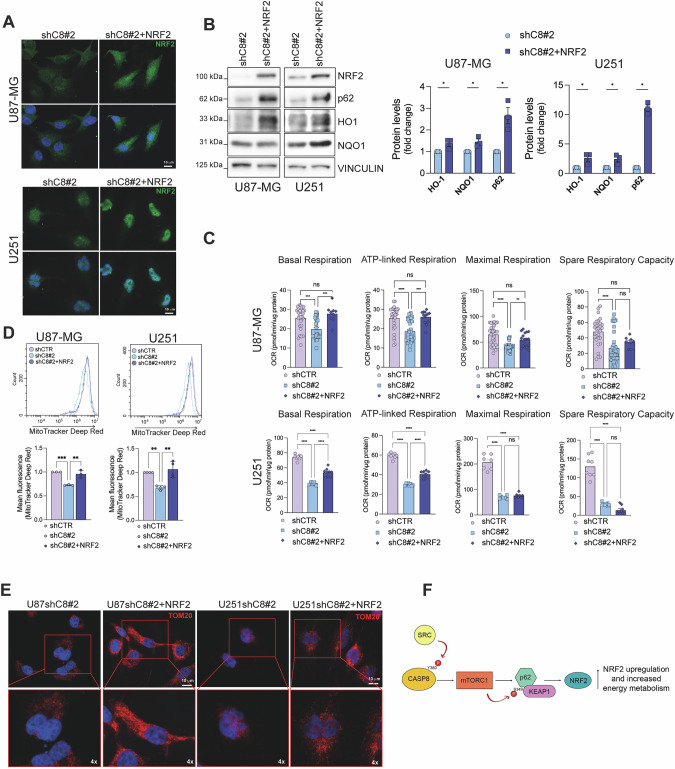
Reconstitution of NRF2 expression rescues the mitochondrial functionality of shC8 cells. **A** Immunofluorescence of U87-MG and U251 shC8#2 and of shC8#2 + NRF2 cells; NRF2 (green) and DNA (Hoechst, blue); **B** Immunoblotting of NRF2 and target proteins p62, HO1 and NQO1 and relative densitometric analyses in the same cells in **A**. Vinculin was used as loading control. **C** Histograms of basal respiration, ATP-linked respiration, maximal respiration and spare respiratory capacity by Seahorse assay showing the oxygen consumption rate (OCR) in U87-MG and U251 shC8#2 and of shC8#2 + NRF2 cells. **D** Cytofluorimetric analyses of active mitochondria upon MitoTracker Deep Red staining in U87-MG and U251 shC8#2 and of shC8#2 + NRF2 cells. **E** Immunofluorescence of U87-MG and U251 shC8#2 and of shC8#2 + NRF2; TOM20 (red) and DNA (Hoechst, blue). **F** Working model. Results represent the mean of at least three independent experiments ( ± SEM). Statistical analyses: paired *t* test (**B**, **D**) and One-way ANOVA test (**C**) (*P < 0.05; ***P < 0.001; ****P < 0.0001).

To unambiguously evaluate the significance of NRF2 downregulation upon Caspase-8 gene silencing, U87shC8 and U251shC8 cells were stably transfected to overexpress the *wild-type* form of NRF2 (U87shC8 + NRF2 and U251shC8 + NRF2) (Fig. 6A). As expected, NRF2 reconstitution sustains the expression of its targets such as NQO1, HO-1 and p62 (Fig. 6B), previously shown to be downregulated in shC8 cells (Fig. 2). In addition, NRF2 expression was sufficient to rescue the mitochondrial functionality of U87shC8 and U251shC8 cells, as shown by mitochondrial parameters that are restored at the level of shCTR cells (Fig. 6C, D) and by immunofluorescence staining for mitochondrial marker TOM20 (Fig. 6E). Overall, these data confirm a role of Caspase-8 on mitochondrial homeostasis directly mediated by NRF2 signaling.

## Discussion

Cancer is considered a genetic disorder characterized by the accumulation of many mutations that ultimately drive the alteration of intra and inter cellular signal transduction [24]. The aberrant activation of oncogenes drives the rewiring of signaling by hijacking the canonical function of many proteins. Although Src kinase is the first oncogene ever identified and its activity is very often aberrantly induced in cancer, the molecular signaling through which constitutive Src activity may sustain tumorigenesis and tumor development have been only partially elucidated [25]. Importantly, the work from our and other laboratories supports a role for Src in the reprogramming of Caspase-8 activity. Src can directly phosphorylate Caspase-8 on Y380 and this phosphorylation impinges on Caspase-8 enzymatic activity, preventing its apoptotic canonical function, therefore justifying the high levels of Caspase-8 in cancer cells [6]. More recently, we and others showed that the aberrant activation of Src and the subsequent phosphorylation of Caspase-8 on Y380 drive also the acquisition by glioblastoma cells of new non canonical functions of Caspase-8 that overall, sustain tumorigenic progression and a poor prognosis [3–5, 7, 8].

NRF2 is a crucial player of oxidative stress response and of metabolic rewiring and, indeed, several reports point to its aberrant activation in cancer progression and to its role in resistance to therapy [21, 26–31]. Consistently, mutations on *KEAP1* or *NFE2L2* genes, that interfere with the ability of KEAP1 to bind and downregulate NRF2 signaling, have been identified in many tumors [31–33]. Remarkably, NRF2 pathway hyperactivation has been reported also independently of the occurrence of genetic mutations, suggesting that aberrant signaling cascades may impinge on this signalling in cancer cells [34]. Importantly, the aberrant functionality of NRF2 in glioblastoma cells has also been recently reported [13, 35, 36]. At the molecular level, it has been shown that a non-canonical molecular mechanism drives sustained p62 phosphorylation on Ser349 which enables p62 to sequester KEAP1 resulting in NRF2 signaling activation [13]. We recently identified Src as a major promoter of mTORC1-dependent phosphorylation of p62 and of NRF2 hyperactivation in glioblastoma cellular models [21].

Being Src a major modulator of both NRF2 signaling and Caspase-8 functionality, we hypothesized a bridging role of the aberrant Src activity connecting Caspase-8 and NRF2.

Here, we discovered by in silico and in vitro experiments a strong impact of Caspase-8 expression on NRF2 expression and signaling in glioblastoma. Indeed, transcriptomic analysis on U87-MG glioblastoma cells genetically silenced or not for Caspase-8 expression highlighted a significant reduction of NRF2 expression and activity in shC8 cells compared to control ones. This finding was also confirmed by proteomic experiments on GBM cellular models.

Indeed, we demonstrate that Caspase-8 expression sustains mTORC1 activity and p62 constitutive phosphorylation on Ser349, thereby resulting in increased p62-KEAP1 interaction thus allowing NRF2 stabilization. Importantly we also confirmed the key role of Src-dependent phosphorylation of Caspase-8 on Y380 in this mechanism.

Of note, proteomic analyses highlighted the ability of Caspase-8 expression to impinge on several signaling pathways including TOR signaling, autophagy, glycolysis and metabolism. Indeed, we demonstrated for the first time that Caspase-8 expression and its phosphorylation on Y380 strongly affect cellular respiration and mitochondrial functionality.

Importantly the reconstitution of NRF2 expression in cells silenced for Caspase-8 is sufficient to rescue this phenotype. While NRF2 is a well-known key player supporting mitochondrial activity and cell metabolism [37, 38], the significance of Caspase-8 in this context deserves further elucidation. Interestingly, recent data from literature highlighted a functional link in esophageal squamous cell carcinoma between Caspase-8 point mutations, frequently identified in this tumor, and the aberrant NRF2 activation that allows cancer cells to cope with oxidative stress and metabolic rewiring [39]. Our findings suggest that the oncogenic activation of Src may represent an alternative mechanism to rewire Caspase-8 function driving its phosphorylation on Y380 that, similarly to what reported for Caspase-8 mutants, promotes mTORC1 activity, p62 phosphorylation on Ser349 and ultimately drives the hyperactivation of NRF2 signaling.

GBM is indeed a very aggressive tumor characterized by poor prognosis. Importantly, upregulation of glycolysis has been identified as a key driver in sustaining the high proliferative rate, aggressiveness, and therapy resistance of GBM [40, 41]. Furthermore, a major issue is related to the heterogeneity of the molecular features of GBM patients. Several previous studies aimed to improve patients’ stratification based on transcriptomic profile identified different GBM subtypes [42, 43]. In searching for amelioration in patients’ management and therapy outcome, it has been recently proposed a robust glycolytic score (GS) model to connect glycolytic signature to other biological features in GBM. Interestingly, GBM with high GS correlate with poor prognosis, and consistently, the mesenchymal subtype exhibits higher GS levels [44]. Of note, Caspase-8 upregulation has been identified as part of the molecular signature of the particularly aggressive mesenchymal subtype [42]. Interestingly, mesenchymal GBMs are also characterized by elevated levels of NRF2 mRNA and protein expression [13]. Furthermore, previous studies identified the tumors that belong this subtype as those that may be more sensitive to the inhibition of Src kinase [45] and we recently reported that Src activation in this context is pivotal to drive NRF2 upregulation [21]. Overall, here we identify an unexpected functional link between Src, Caspase-8 and NRF2 which may be particularly relevant for mesenchymal GBM metabolic dependencies. Of note, bioinformatic analyses of TCGA data suggest that Caspase-8 and NRF2 expression positively correlate also in other tumors.

Lastly, Caspase-8 expression and its phosphorylation may therefore represent novel markers to identify those tumors characterized by NRF2-dependent metabolic rewiring, paving the way for discovery new therapeutic vulnerabilities.

## Materials and methods

### Cell culture

All GBM cell lines were cultured in DMEM (Dulbecco’s Modified Eagle’s Medium - high glucose, Sigma-Aldrich) supplemented with 10% fetal bovine serum (FBS, Sigma-Aldrich), L-glutamine (2 mM, Sigma-Aldrich), 100 U/ml penicillin and 100 mg/ml streptomycin (P/S, Sigma-Aldrich). GBMSC83 (mesenchymal GBM cellular model) shCTR (pLKO) and shC8 previously generated [8] were cultured as neurospheres in non-adherent conditions in DMEM/F12 (Dulbecco’s modified Eagle’s media/F12, 1/1, Thermo Fisher) supplemented by B27 Supplement [50x], EGF (20 ng/ml), and hβFGF (10 ng/ml) as previously described [46, 47]. All GBM cells were routinely tested negative for *Mycoplasma* contamination. The following sequences were used for stable retroviral Caspase-8 silencing (shC8#1) in U87-MG and U251 cell lines: shC8#1:5′-ATCACAGACTTTGGACAAA-3′; as control sequence, shCTR: 5′- CTATAACGGCGCTCGATAT-3′ [7]. For stable lentiviral Caspase-8 silencing (shC8#2) E42 clone ID TRCN0000003579 commercially available (Open Biosystem, Dharmacon, now Horizon) was used according to the manufacturer’s instructions. The original targeted DNA sequence is 5′-GCCTTGATGTTATTCCAGAGA-3′. For the stable reconstitution of Caspase-8-WT and the mutant Caspase-8-Y380F, shC8#2 cell lines were infected with lentiviral pLV-EF1a IRES vector encoding for Caspase-8- WT or Caspase-8-Y380F carrying the mutations that do alter aminoacidic sequence but confers resistance to the above interference 5′- GCacTtATGcTtTTCCAacGt-3′. Stable cell lines U87shC8#2 and U251shC8#2, overexpressing pCDNA3.1-FLAG-NRF2^WT^ (#36971, Addgene), were generated by transfection using polyethylenimine (PEI) (Tebu-Bio), following the manufacturer’s instructions. Cells were maintained and selected with 50 µg/ml Zeocin (Thermo Fisher) for 2 weeks. Stable cell lines U87shCTR, U87shC8, U251shCTR, U251shC8 (#1), overexpressing the autophagic probe GFP-LC3-RFP-LC3ΔG, were generated by lentiviral infection with pMRX-IP-GFP-LC3-RFP-LC3ΔG vector [16].

For transient silencing, U87-MG cells were transfected with polyethylenimine (PEI) (Tebu-Bio) by using control siRNA and Caspase-8 siRNA (Santa Cruz Biotechnology; sc-29930) according to the manufacturer’s instructions.

### Antibodies and other reagents

Primary antibodies used are as follows: anti-Caspase-8 (5F7) (MBL International, 1:1000); anti-NRF2 (Cell Signaling Technology 1:1000, D1Z9C); anti-p62 (SQSTM1) (MBL International 1:5000, PM045); anti-phospho-p62 (SQSTM1) (Ser349) (MBL International 1:1000, PM074); anti-GAPDH (Cell Signaling Technology 1:5000, D16H11); anti-heme oxygenase 1 (A-3) (Santa Cruz Biotechnology 1:1000, sc-136960; anti-NQO1 (A-180) (Santa Cruz Biotechnology 1:1000, sc-32793); anti-GPX4 (Santa Cruz Biotechnology, 1:1000); anti-mTOR (Santa Cruz Biotechnology 1:1000, sc-517464); anti-phospho-mTOR (Ser2481) (Santa Cruz Biotechnology 1:1000, sc-293132); anti-p70S6K (Cell Signaling Technology, 1:2000); anti-phospho-p70S6K (Thr389) (Cell Signaling Technology, 1:1000); anti-Vinculin (Cell Signaling Technology 1:5000, E1E9V); anti-KEAP1 (Santa Cruz Biotechnology, 1:1000); anti-LC3 (MBL International 1:5000); anti-TOM20 (Santa Cruz Biotechnology, 1:1000); anti-Lamin A/C (Santa Cruz Biotechnology 1:1000; sc-376248;); anti-TFEB (Cell Signaling Technology, 1:1000; 4240S); anti-β-Actin (Cell Signaling Technology, 1:5000; 3700 T), anti-GR (Santa Cruz Biotechnology, 1:1000; sc-133245); anti-ATPB (Abcam, 1:1000; ab-14730); anti-VDAC (Santa Cruz Biotechnology, 1:1000; sc-390996). Chloroquine was purchased from Sigma-Aldrich.

### Immunofluorescence

Cells were plated on coverslips and growth at 37 °C and 5% CO_2_. Before the staining, cells were washed with 1x phosphate buffer saline (PBS) and fixed in 4% PFA for 15 minutes at room temperature, permeabilized with PSB/Triton X-100 0,3%, blocked with BSA 3% solution in PBS for 1 h and then incubated overnight with primary antibody NRF2, 1:100; KEAP1, 1:50; p62: 1:1,000; TFEB 1:50; TOM20 1:500) in a humid chamber at 4 °C. Secondary antibody (1:500; Thermo Fisher Scientific) were applied for 1 h at RT, and nuclei were stained with Hoechst 33342 (Thermo Fisher Scientific) for 15 min. Images were acquired by fluorescence microscopy (ZEISS) and processed with Fiji version 2.3. Staining intensities were analyzed by using Fiji software (ImageJ) to obtain the nuclear/cytoplasmatic ratio.

Confocal microscopy experiments were performed by using LSM800 microscope (ZEISS) equipped with a 63x oil objective and with ZEN blue imaging software. For colocalization studies, at least three slices were taken at a Z-stack distance of 0.3 μm. After acquisition, images were imported into Fiji for analysis and quantification of colocalizing particles between two channels by using the open-source plugin ComDet 0.5.2. Colocalization parameters were as follows: maximum distance between the center of two particles ≤4 pixels; particle size ≥3; intensity threshold = 5.

### Protein extracts, nuclei/cytoplasm fractionation, immunoprecipitation and immunoblotting analyses

Total cell extracts were prepared in RIPA Buffer (50 mM Tris-HCL pH 8.0, 150 mM NaCl, 1% NP40, 12 mM sodium deoxycholate) supplemented with 1 mM phenyl-methyl-sulfonyl-fluoride, 25 mM NaF, 1 mM sodium orthovanadate, 25 mM β-glycerol-phosphate, 10 mg/ml TPCK, 10 mg/ml cocktail inhibitor (Sigma-Aldrich, P2714). Lysates were incubated for 30 min on ice and centrifugated 12,000 g at 4 °C for 20 min. For nuclei and cytoplasm fractionation, cells were lysed in Buffer A (10 mM Hepes pH 7.9, 10 mM KCl, 1.5 mM MgCl2, 0.5 mM DTT), supplemented as RIPA buffer, for 20 min on ice. NP-40 was then added to a final concentration of 0.1%. Next, nuclei were separated from the cytoplasm by centrifugation at 12,000 g at 4 °C for 30 sec. The cytoplasm was harvested and the nuclear pellet was lysed in Buffer A supplemented with 0.05% NP-40 for 10 min on ice, then sonicated and centrifuged at 12,000 g for 20 min. For immunoprecipitation experiments, total cell extracts were incubated with the primary antibody overnight on a rotating wheel at 4 °C, followed by 45 min incubation with Protein G beads (Invitrogen). The complex was washed four times in ice-cold PBS 1X, denatured for 5 min at 95 °C. For immunoblotting, 10–50 µg of proteins were separated by SDS–PAGE, blotted onto nitrocellulose membrane, and detected with specific antibodies.

### Reverse Transcription and real-time polymerase chain reaction (qPCR)

Cells were homogenized in TRI Reagent (Thermo Fisher Scientific), and RNA was extracted in accordance with the manufacturer’s protocol. 1 μg of total RNA was retrotranscribed in cDNA using the SensiFAST cDNA Synthesis KIT (Bioline). RT–qPCR was performed using the SensiFAST Syber Low-ROX kit (Bioline) and QuantStudio 3 RT–qPCR (Applied Biosystems). Data were analyzed by using the second-derivative maximum method. The fold changes in mRNA levels were determined relative to actin, a control after normalizing to the internal standard. Primers used for each gene are listed below:

ACTIN 5’-GGCCGAGGACTTTGATTGCA-3’5’-GGGACTTCCTGTAACAACGCA-3’

HMOX-1 5’-CACAGCCCGACAGCATGCCC-3' 5’-GCCTTCTCTGGACACCTGACCCT-3’

NQO1 5’-GGTTTGGAGTCCCTGCCATT-3’ 5’-CCTTCTTACTCCGGAAGGGTC-3’

GCLC 5’-CGCACAGCGAGGAGCTTCGG-3’ 5’-CTCCACTGCATGGGACATGGTGC-3’ 31

SQSTM1 5’-GGGAAAGGGCTTGCACCGGG-3’ 5’-CTGGCCACCCGAAGTGTCCG-3’

NFE2L2 5’-TTCCCGGTCACATCGAGAG-3’ 5’-TCCTGTTGCATACCGTCTAAATC-3’

### Proteomic and (phospho)proteomic sample preparation

U87shCTR and shC8 cells were seeded (500.000 cells for each p100 dish) and the day after they were washed in TBS1X and scraped on ice (with 1 ml of ice-cold TBS1X/p100) using a cell scraper. Then cells were collected in protein LoBind Tube 1.5 ml (Eppendorf) and centrifuged (4 °C, 2300 rpm, 5 minutes). Cell pellets were snap-frozen in liquid nitrogen and stored at −80 °C until lysis.

Proteins were extracted in lysis buffer containing 10% ACN, 60 m M TEAB, 5 m M TCEP and 25 m M CAA and incubated at 76 °C for 20 minutes with mixing. Next, samples were sonicated in bioruptor for 15 cycles at high intensity 30 s on/30 s off. 1 mg of lysate was digested with trypsin and Lys-C (10 μg each) overnight at 37 °C. The digestion was stopped adding 1% of pure acid formic and samples were centrifuged at 5000xg for 3 minutes. Phosphorylated peptides were enriched by using Zr-IMAC beads previously washed with 0.1 M glycolic acid+80%ACN + 5% formic acid three times. For each sample 500 μg of digested proteins were incubated with 40 μl of magnetic beads and incubated at room temperature for 20 minutes with constant mixing (1500 rpm). Peptides-beads complexes were isolated on a magnetic separator and sequentially washed with i) 0.1 M glycolic acid+80%ACN + 5% formic acid ii) 80%ACN + 1%formic acid and iii) 10%ACN + 0.2%TFA. Finally, phosphorylated peptides were eluted with 1% NH4OH and the collected supernatant was resuspended in 50 μl of 10%TFA. Before the phosphopeptide enrichment, 50 μg of the digested peptides were used for the total proteome analysis. inStageTip (iST) method was used for the proteome and phosphoproteome preparation [48]. Briefly, SDBRPS tips were washed with i) 100 µl acetonitrile (ACN), ii) 100 µl of 30% methanol and 1% TFA and iii) 150 µl of 0.2% TFA by centrifuging tips at 1000 xg for 3 minutes. Samples were loaded onto equilibrated columns and spin at 1000 xg for 10 minutes. SDBRPS tips were washed with i) 100 µl of 1% TFA in ethyl acetate, ii) 100 µl of 1% TFA in isopropanol and iii) 0.2% TFA. For protein elution, we used a buffer containing 80% ACN, 5% NH4OH in MilliQ water. Samples were centrifuged at 1000 xg for 4 minutes and concentrated by SpeedVac at 45° for ~45 minutes. Finally, samples were resuspended in 10 μl of a buffer additioned with 2% ACN and 0.1% TFA.

### Mass spectrometry analysis

The peptides and phosphopeptides were separated on a reverse-phase column (50 cm, packed in-house with 1.9-mm C18-Reprosil-AQ Pur reversed-phase beads) (Dr. Maisch GmbH). For single-run proteome analysis, separation occurred over 120 minutes, while for phosphoproteome analysis, it extended to 140 minutes. Following elution, the peptides were subjected to electrospray ionization and analyzed via tandem mass spectrometry using an Orbitrap Exploris 480 instrument (Thermo Fisher Scientific). The instrument operated by alternating between a full scan and multiple high-energy collision-induced dissociation (HCD) fragmentation scans, resulting in a total cycle time of up to 1 second.

### Proteome and phosphoproteome data processing

Raw files were analyzed using the Spectronaut software. MS/MS spectra were searched against the Homo sapiens UniProtKB FASTA database (September 2014), with an FDR of <1% at the level of proteins, peptides and modifications. Enzyme specificity was set to trypsin, allowing for cleavage N-terminal to proline and between aspartic acid and proline. The search included cysteine carbamidomethylation as a fixed modification. Variable modifications were set to N-terminal protein acetylation and oxidation of methionine as well as phosphorylation of serine, threonine tyrosine residue (STY) for the phosphoprotemic samples.

### Proteome and phosphoproteome bioinformatics data analysis

Bioinformatic analysis was performed in the Perseus software environment [49]. Statistical analysis of both the proteome and phosphoproteome was executed on logarithmized intensities of quantified values across experimental conditions. Normalization of phosphopeptide intensities was performed by subtracting the median intensity of each sample. To identify significantly modulated proteins and phosphopeptides between conditions, a Student t-test with a permutation-based false discovery rate (FDR) cutoff of 0.05 and S0 = 0.1 was employed. Categorical annotation, such as GO biological process (GOBP), KEGG pathways and kinase substrate motifs (extracted from HPRD), was added in Perseus. Proteomic data have been submitted to PRIDE (dataset identifier PXD060920).

### Bioenergetic analysis

The bioenergetic analyses were performed using the Seahorse XF96e Analyzer (Seahorse Bioscience Agilent, Santa Clara, CA, USA). Briefly, cells were seeded at a density of 12×10^3^ live cells per well on Seahorse XF96 microplates (Agilent Technologies, Santa Clara, CA, USA, cat. no. 103794-100).

The Cell Mito Stress Test was conducted according to the manufacturer’s instructions to evaluate mitochondrial function by monitoring the oxygen consumption rate (OCR) in real time. Various inhibitors were sequentially injected to perturb the electron transport chain complexes: oligomycin an ATP synthase inhibitor, to determine ATP production-coupled respiration; carbonyl cyanide-p-trifluoromethoxyphenylhydrazone (FCCP), an uncoupler, to measure the maximal respiration rate; and a combination of rotenone and antimycin A, inhibitors of complexes I and III respectively, to assess non-mitochondrial respiration. The concentrations of the compounds used were as follows: 1.5 µM oligomycin A (Merck KGaA, Darmstadt, Germany, cat. no. 75351), 1 µM FCCP (Merck KGaA, cat. no. C2920), 0.5 µM rotenone (Merck KGaA, cat. no. R8875), and 0.5 µM antimycin A (Merck KGaA, cat. no. A8674).

The assay enabled the determination of key parameters of mitochondrial function, including basal respiration (baseline OCR before oligomycin addition), ATP-linked respiration (difference between basal respiration and the minimal respiration after oligomycin), and maximal respiration (OCR following FCCP addition). After the assay, cells were lysed with 10 µL of 0.1% SDS in water, and protein quantitation was performed to normalize OCR data, which were expressed as pmol O_2_/min/μg protein. The data were analyzed using the Seahorse Analytics online platform (Agilent Technologies, https://seahorseanalytics.agilent.com/) or XFe Wave software (Santa Clara, CA, USA).

### Flow cytometry analysis

For mitochondrial content and activity assays, cells were incubated with 50 nM of MitoTracker Green or Deep Red dyes (Invitrogen), respectively, in serum-free medium for 20 minutes at 37 °C. Cells were then collected and centrifuged at 300 g for 5 min at 4 °C and resuspended in Hanks′ Balanced Salt solution (HBSS, Sigma-Aldrich). Fluorescence intensity was evaluated with CytoFLEX S (Beckman Coulter) instrument. Unstained samples were used as control. Quality control was evaluated using CytoFLEX Daily QC Fluorospheres (Beckman Coulter). FCS files were analyzed using CytExpert version 2.2 software (Beckman Coulter).

### DNA isolation and real time qPCR

Mitochondrial and genomic DNA were isolated according to Miller et al. [50]. Mitochondrial DNA content expression levels were determined by Real Time qPCR reactions using a Light Cycler 480 SYBR Green System (Roche ETC). Cp values were calculated using the ‘second derivative max’ algorithm of the Lightcycler software. Relative mitochondrial DNA content was normalized to the genomic genes β-actin. Results, where not specifically expressed, were reported as the ratio between the average of values from different cellular models, normalized to the average values of genomic genes or the housekeeping gene. Primers sequences are listed below:

- ND5: R: TGAGTGGAGTAGGGCTGAGAC F: CTAGCAGCAGCAGGCAAAT
- ND2: R: GCTTGTTTCAGGTGCGAGAT F: CTACCGCATTCCTACTACTCA
- COX: R; TGAAATTGATGGCCCCTAAG F: CCCTCCCTTAGCAGGGAAC

### Statistical analysis

All experiments were replicated at least three times (biological replicates) and data were presented as mean ± SEM, as indicated in the figure legends. The significance of the differences between populations of data was assessed according to the two-tailed *t* test (independent samples) with level of significance of at least *P* ≤ 0.05. For immunofluorescence analysis, unpaired *t* test was used. For multiple comparisons, we used one-way ANOVA test followed by Kruskal–Wallis test. All the statistical analyses were performed using GraphPad Prism 10 software.

## Supplementary information

Supplementary Figure Legends

Original Wester Blots

Table S1_Dataset 1_Proteomic dataset

Table S2_Dataset 2_Phosphoproteomic dataset

TableS3

TableS4

## Data Availability

All data needed to evaluate the conclusions in the paper are present in the paper and/or the Supplementary Materials. Additional data related to this paper may be requested from the authors.
