# Supplementary material for: Caspase-8 expression and its Src dependent phosphorylation on Tyrosine 380 triggers NRF2 signaling activation in glioblastoma
Source: Cell Death Differ. 2025 Oct 6;32(12):2355–67. doi: 10.1038/s41418-025-01542-3 (PMC12669649; doi:10.1038/s41418-025-01542-3)
Supplement: Supplementary file 1 — Supplementary Figure Legends [file 41418_2025_1542_MOESM1_ESM.pdf]

Supplementary Figure S1

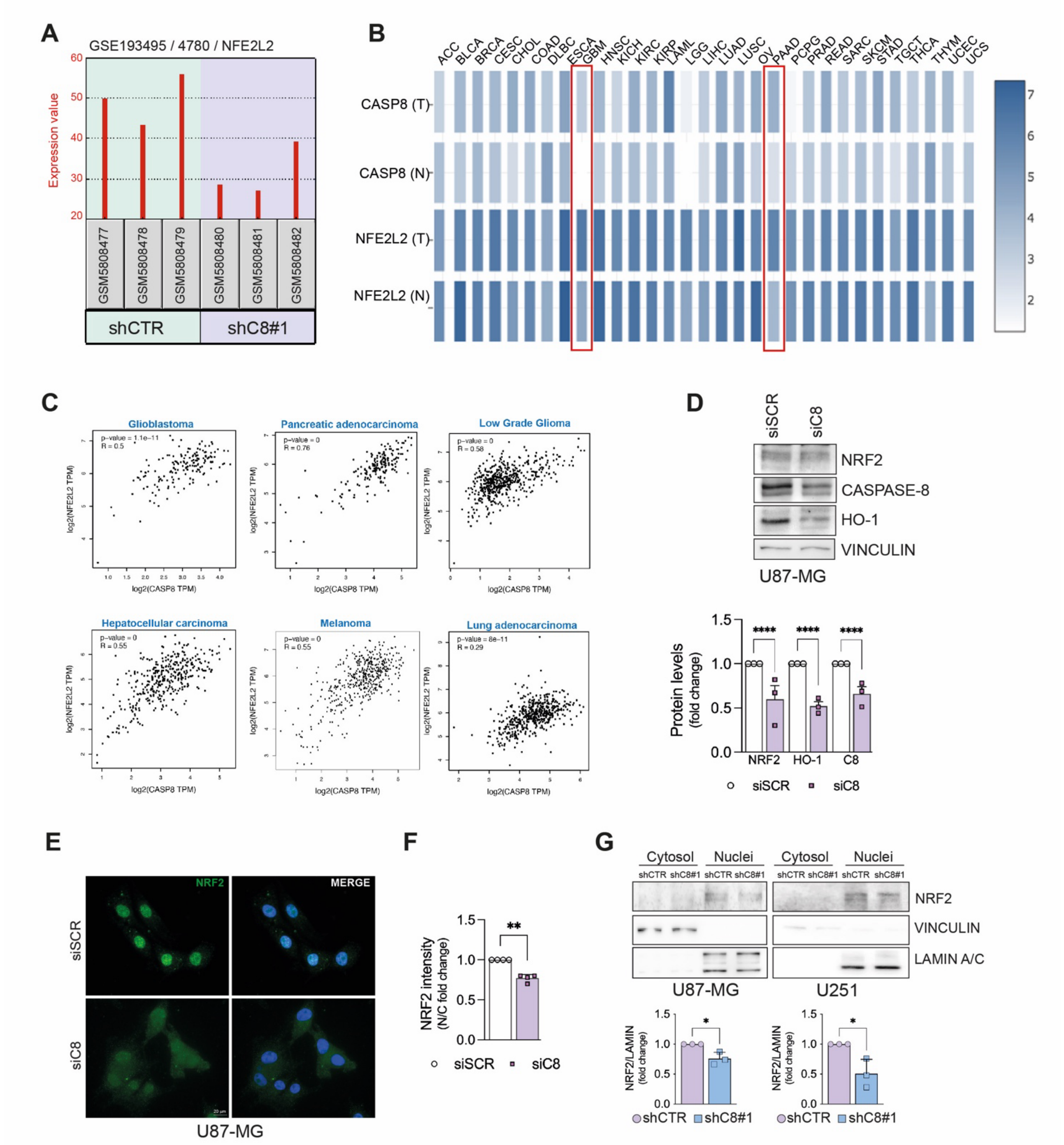

Supplementary Figure S1. A) Bar Plot of the Transcripts Per Kilobase Million (TPM) normalized expression values of NFE2L2 gene across 3 U87shCTR samples and 3 U87shC8#1 samples, obtained from GEO dataset (GSE193495). **B**) Expression matrix plots based on *CASP8* and *NFE2L2* genes obtained by Multiple Gene Comparison tool (GEPIA2). The density of color in each block represents the median expression value of a gene in each tissue, normalized by the maximum median expression value across all blocks. Red boxes highlight tumors (glioblastoma, GBM, and pancreatic adenocarcinoma, PAAD) in which the expression of *CASP8* and *NFE2L2* is significantly higher, compared to normal tissues. **C**). Scatter plot showing positive

Correlation of *CASP8* and *NFE2L2* gene expression in glioblastoma, pancreatic adenocarcinoma, low grade glioma, hepatocellular carcinoma, melanoma and lung adenocarcinoma, obtained by Multiple Gene Comparison tool (GEPIA2). **D**) Immunoblotting and relative densitometric analyses of NRF2, Caspase-8 and HO-1 in U87-MG cells transiently silenced (siC8) or not (siSCR), for 24h. Vinculin was used as loading control. Results represent the mean at least of three independent experiments (± SEM). Statistical analysis: paired *t* test. ****P<0.0001. **E-F**) Immunofluorescence and relative quantification reported as the ratio between nuclear and cytosolic fluorescence intensity (N/C) of NRF2 staining in U87-MG cells transiently silenced (siC8) or not (siSCR), for 24h. NRF2 (green) and DNA (Hoechst, blue). Results represent the mean at least of three independent experiments (± SEM). Statistical analysis: paired *t* test. **P<0.01. **G**) Immunoblotting and relative densitometric analyses of NRF2 cytosolic and nuclear fractions in U87-MG and U251 shCTR and shC8#1 cells. Vinculin and Lamin A/C were used as loading and quality controls. Results represent the mean at least of three independent experiments (± SEM). Statistical analyses: paired *t* test. *P<0.05.

Supplementary Figure S2

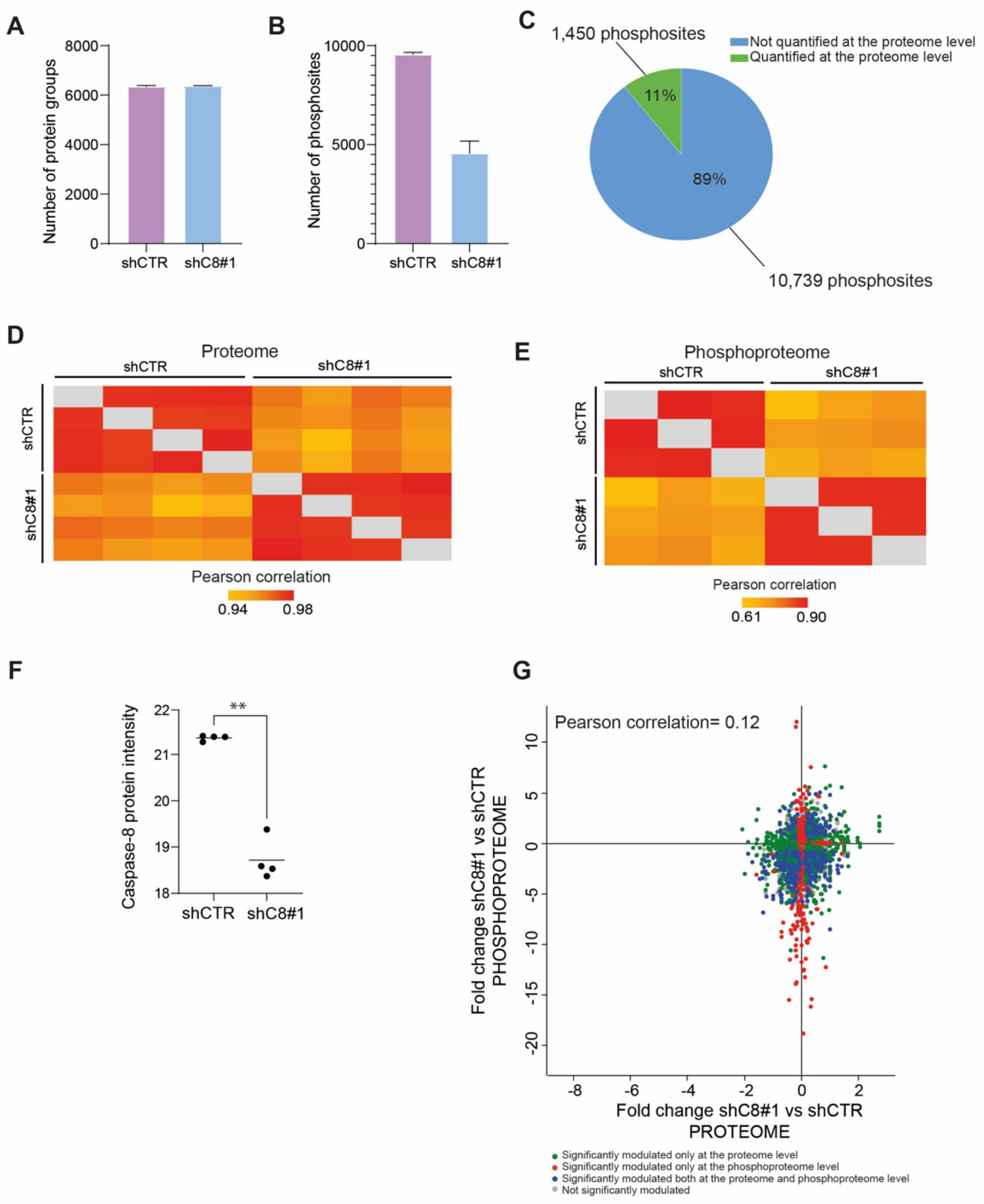

Supplementary Figure S2. A) Bar plots reporting the number of protein groups quantified in the U87shCTR and U87shC8#1 and samples. **B**) Bar plots reporting the number of phosphosites (B) quantified in the U87shCTR and U87shC8#1 and samples. **C**) Pie chart reporting the percentage and the number of the phosphosites with or without the quantification of the corresponding total protein level. **D-E**) Correlation matrix of the Pearson correlation coefficient for normalized log2 protein (D) and phosphosites intensity (E). **F**) Caspase-8 protein expression levels quantified in the U87shC8#1 and U87shCTR samples. **G**) The modulation of the proteome and the phosphoproteome were compared and represented in the scatterplot in which each dot represents one protein. Proteins and phosphorylation sites were considered modulated between U87shC8#1 and U87shCTR samples according to the t test analysis (p-Value <0.05).

Supplementary Figure S3

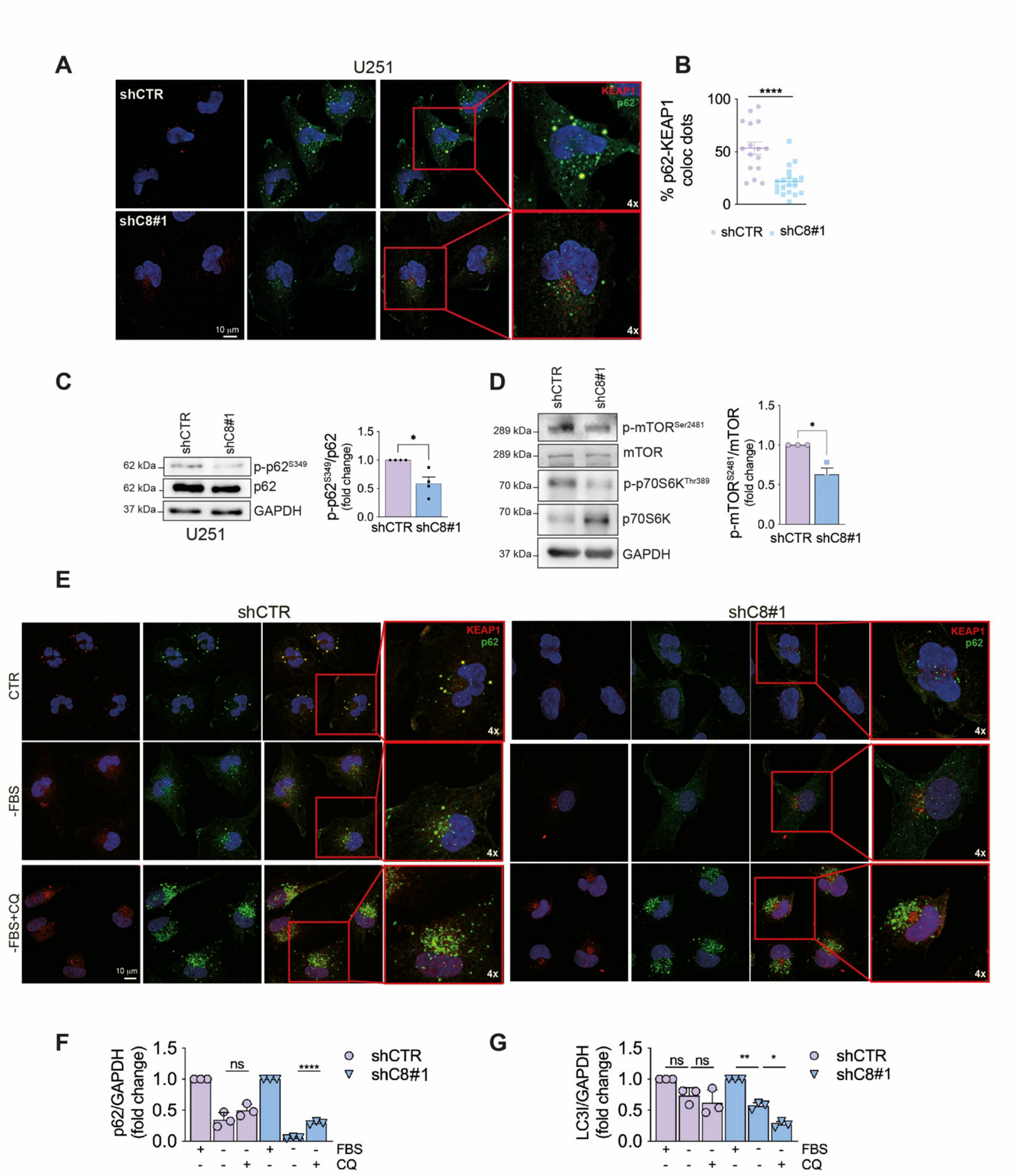

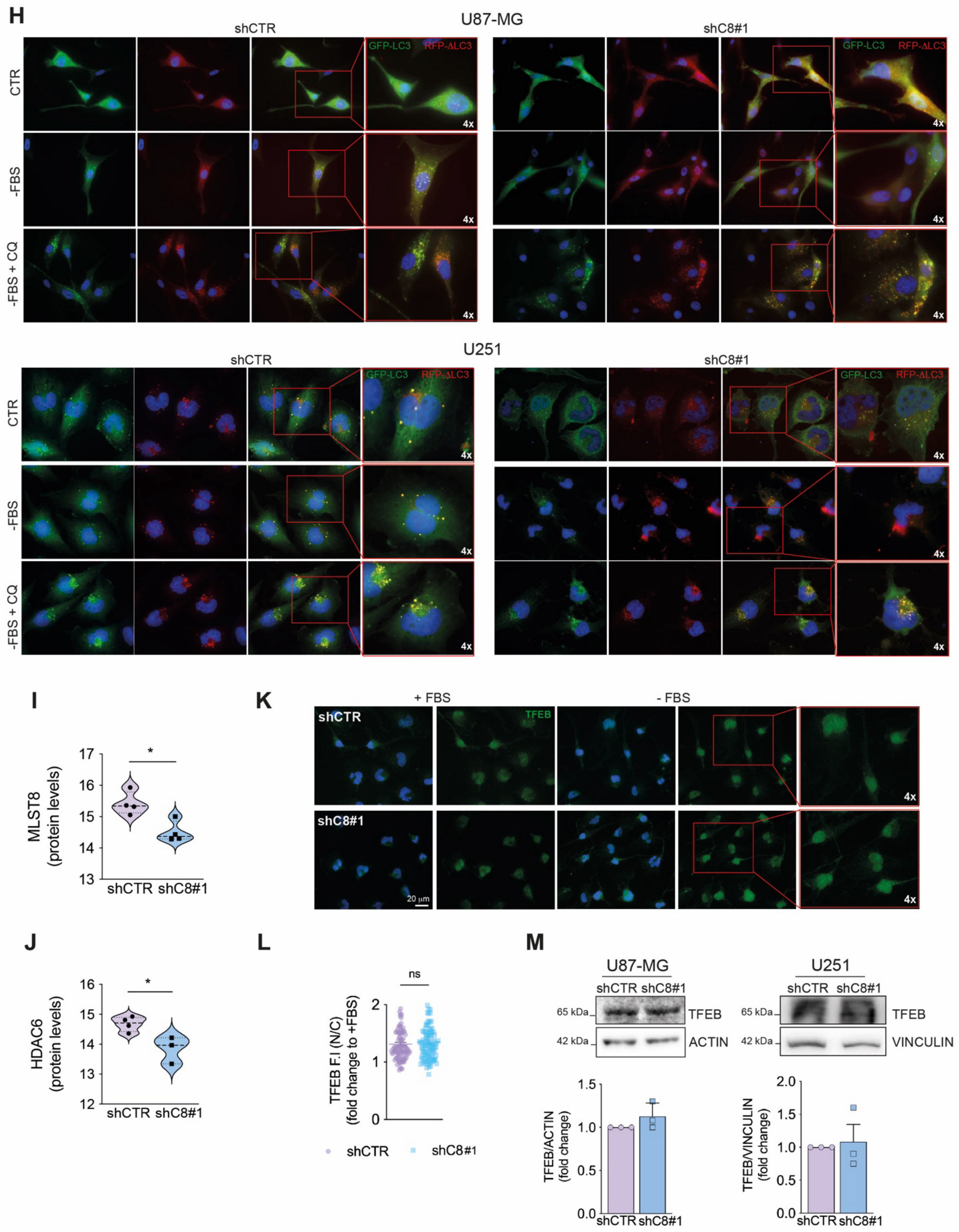

H
U87-MG
shCTR
shC8#1
GFP-LC3
RFP-ΔLC3
CTR
-FBS
-FBS + CQ
4x
U251
I
MLST8
(protein levels)
shCTR shC8#1
K
+ FBS
- FBS
TFEB
20 μm
J
HDAC6
(protein levels)
L
TFEB F.I (N/C)
(fold change to +FBS)
ns
M
65 kDa
42 kDa
TFEB
ACTIN
VINCULIN
TFEB/ACTIN
(fold change)
TFEB/VINCULIN
(fold change)

Supplementary Figure S3. A) Confocal microscopy analyses of U251shCTR and U251shC8#1 cells; p62 (green), KEAP1 (red), DNA (Hoechst, blue); 4x digital magnification showing merged signals. **B**) Quantification of colocalizing p62/KEAP1 dots in U251shCTR and U251shC8#1 cells. Results represent the mean at least of three independent experiments (± SEM). Statistical analysis: unpaired *t* test. ****P<0.0001. **C**) Immunoblotting and relative densitometric analysis of p-p62^{S349} normalized on total p62 in U251shCTR and U251shC8#1 cells. GAPDH was used as loading control. Results represent the mean at least of three independent experiments (± SEM). Statistical analysis: paired *t* test. *P<0.05. **D**) Immunoblotting of p-mTOR^{Ser2481}, mTOR, p-p70S6K^{Thr389} and p70S6K in U251shCTR and U251shC8# cells and relative densitometric analysis of p-mTOR^{Ser2481} normalized on total mTOR. GAPDH was used as loading control. Results represent the mean at least of three independent experiments (± SEM). Statistical analysis: paired *t* test. *P<0.05. **E**) Confocal microscopy analyses in U251shCTR and U251shC8#1 cells upon serum deprivation (-FBS) for 2 hours and treated with chloroquine (CQ) 10 μM for 16 hours; p62 (green), KEAP1 (red), DNA (Hoechst, blue); 4x digital magnification showing merged signals. **F**) Densitometric analysis of p62 normalized on GAPDH in U87shCTR and U87shC8#1 cells upon serum deprivation (-FBS) for 2 hours and chloroquine (CQ) 10 μM for 16 hours. Results represent the mean at least of three independent experiments (± SEM). Statistical analysis: paired *t* test. ns: not significant, ****P<0.0001. **G**) Densitometric analyses of LC3I normalized on GAPDH in U87shCTR and U87shC8#1 cells upon serum deprivation (-FBS) for 2 hours with or without chloroquine (CQ) 10 μM for 16 hours. Results represent the mean at least of three independent experiments (± SEM). Statistical analysis: paired *t* test. ns: not significant, *P<0.05, **P<0.01. **H**) Fluorescence microscopy analyses of U87-MG and U251 shCTR and shC8#1 cells stably expressing GFP-LC3-RFP-LC3ΔG probe, upon serum deprivation (-FBS) for 2 hours and chloroquine (CQ) 10 μM for 16 hours. GFP-LC3 (green), RFP-ΔLC3 (red), DNA (Hoechst, blue); 4x digital magnification showing merged signals. The images were acquired by ApoTome module. **I**) MLST8 protein expression levels quantified in the U87shCTR and U87shC8#1 samples. *P<0.05. **J**) HDAC6 protein expression levels quantified in the U87shCTR and U87shC8#1 and samples. *P<0.05. **K**) Immunofluorescence of TFEB in U251shCTR and U251shC8#1 cells upon serum deprivation (-FBS); TFEB (green), DNA (Hoechst, blue); 4x digital magnification showing merged signals. **L**) Quantification of TFEB staining reported as the ratio between nuclear and cytosolic fluorescence intensity (N/C) in U251shCTR and U251shC8#1 cells. Results represent the mean at least of three independent experiments (± SEM). Statistical analysis: unpaired *t* test. **M**) Immunoblotting and relative densitometric analyses of TFEB in U87-MG and U251 shCTR and shC8#1 cells. Actin and Vinculin were used as loading controls. Results represent the mean of three independent experiments (± SEM). Statistical analysis: unpaired *t* test.

Supplementary Figure S4

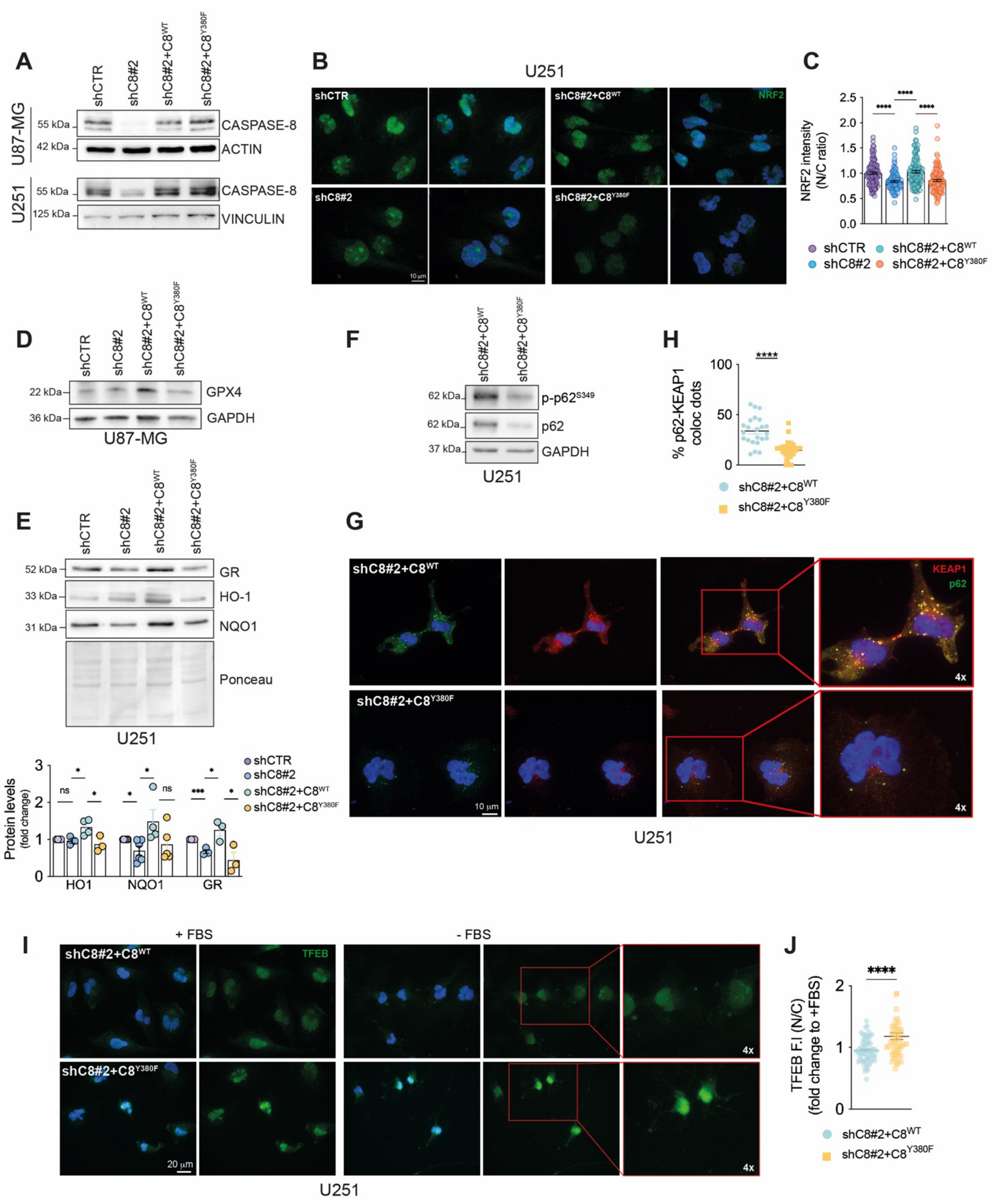

Supplementary Figure S4. **A**) Immunoblotting of Caspase-8 in U87-MG and U251 shCTR, shC8#2, shC8#2+C8^{WT} and shC8#2+C8^{Y380F} cells. Actin and Vinculin were used as loading control. **B**) Immunofluorescence of NRF2 in U251shCTR, U251shC8#2, U251shC8#2+C8^{WT} and U251shC8#2+C8^{Y380F} cells. NRF2 (green) and DNA (Hoechst, blue). **C**) Quantification of NRF2 staining of B) reported as the ratio between nuclear and cytosolic fluorescence intensity (N/C). Results represent the mean at least of three independent experiments (± SEM). Statistical analysis: One-way ANOVA test. **** P<0.0001. **D**) Immunoblotting of GPX4 in U87shCTR, U87shC8#2, U87shC8#2+C8^{WT} and U87shC8#2+C8^{Y380F} cells. GAPDH was used as loading control. **E**) Immunoblotting and relative densitometric analyses of GR, HO-1 and NQO1 in U251shCTR, U251shC8#2, U251shC8#2+C8^{WT} and U251shC8#2+C8^{Y380F} cells. Ponceau staining was used as loading control. Results represent the mean at least of three independent experiments (± SEM). Statistical analysis: Multiple t test. *P<0.05, ***P<0.001. **F**) Immunoblotting of p-p62^{S349} and p62 in U251shC8#2+C8^{WT} and U251shC8#2+C8^{Y380F} cells. GAPDH was used as loading control. **G**) Confocal microscopy analyses of colocalizing dots in U251shC8#2+C8^{WT} and U251shC8#2+C8^{Y380F} cells; p62 (green), KEAP1 (red), DNA (Hoechst, blue); 4x digital magnification showing merged signals. **H**) Quantification of colocalizing p62/KEAP1 dots in U251shC8#2+C8^{WT} and U251shC8#2+C8^{Y380F} cells. Results represent the mean at least of three independent experiments (± SEM). Statistical analysis: unpaired *t* test. ****P<0.0001. **I**) Representative immunofluorescence of TFEB staining in U251shC8#2+C8^{WT} and U251shC8#2+C8^{Y380F} cells upon serum deprivation (-FBS); TFEB (green), DNA (Hoechst, blue); 4x digital magnification showing merged signals. **J**) Quantification of TFEB staining reported as the ratio between nuclear and cytosolic fluorescence intensity (N/C) in U251shC8#2+C8^{WT} and U251shC8#2+C8^{Y380F} cells. Results represent the mean at least of three independent experiments (± SEM). Statistical analysis: unpaired *t* test. ****P<0.0001.

Supplementary Figure S5

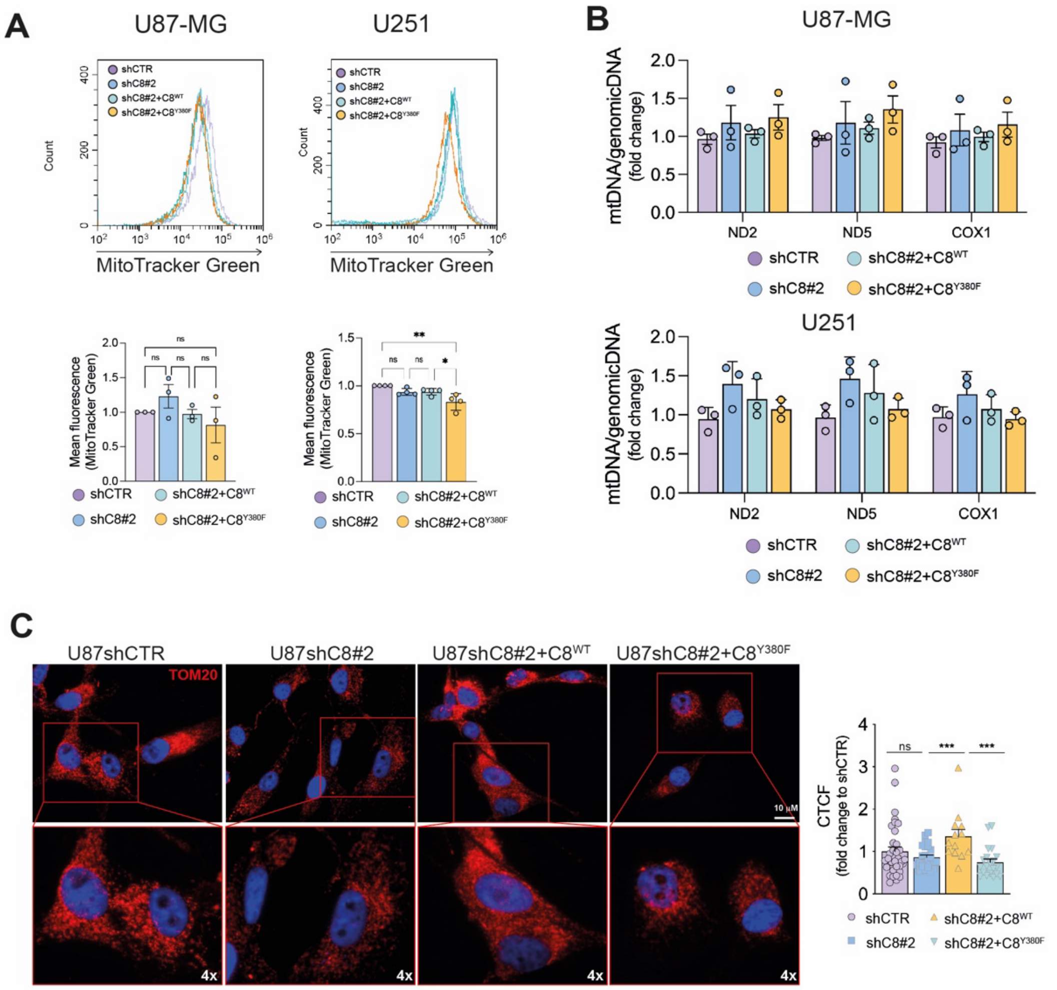

Supplementary Figure S5. A) Cytofluorimetric analyses of mitochondrial content upon MitoTracker Green staining and relative quantification in U87-MG and U251 shCTR, shC8#2, shC8#2+C8^{WT} and shC8#2+C8^{Y380F} cells. Results represent the mean of at least three independent experiments (± SEM). Statistical analysis: One-way ANOVA test. ns: not significant, *P<0.05, **P<0.01. **B**) RT-qPCR of mitochondrially encoded genes ND2, ND5, COX1 normalized on genomicDNA in U87-MG and U251 shCTR, shC8#2, shC8#2+C8^{WT} and shC8#2+C8^{Y380F} cells. Results represent the mean of three independent experiments (± SEM). **C**) Immunofluorescence and relative histograms of corrected total cell fluorescence (CTCF) of TOM20 (red) in U87shCTR, U87shC8#2, U87shC8#2+C8^{WT} and U87shC8#2+C8^{Y380F} cells. DNA (Hoechst, blue). 4x digital magnification showing merged signals. Results represent the mean of at least three independent experiments (±SEM). Statistical analyses: unpaired t test. ***P< 0.001.
