# Supplementary material for: Caspase-8 expression and its Src dependent phosphorylation on Tyrosine 380 triggers NRF2 signaling activation in glioblastoma
Source: Cell Death Differ. 2025 Oct 6;32(12):2355–67. doi: 10.1038/s41418-025-01542-3 (PMC12669649; doi:10.1038/s41418-025-01542-3)

Original WB - Figure 1

Figure 1C

U87-MG

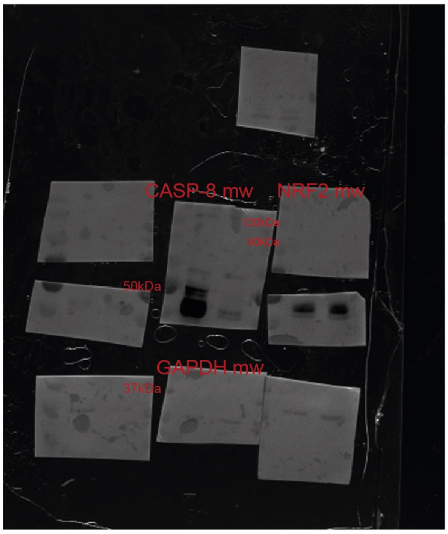

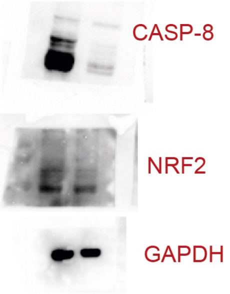

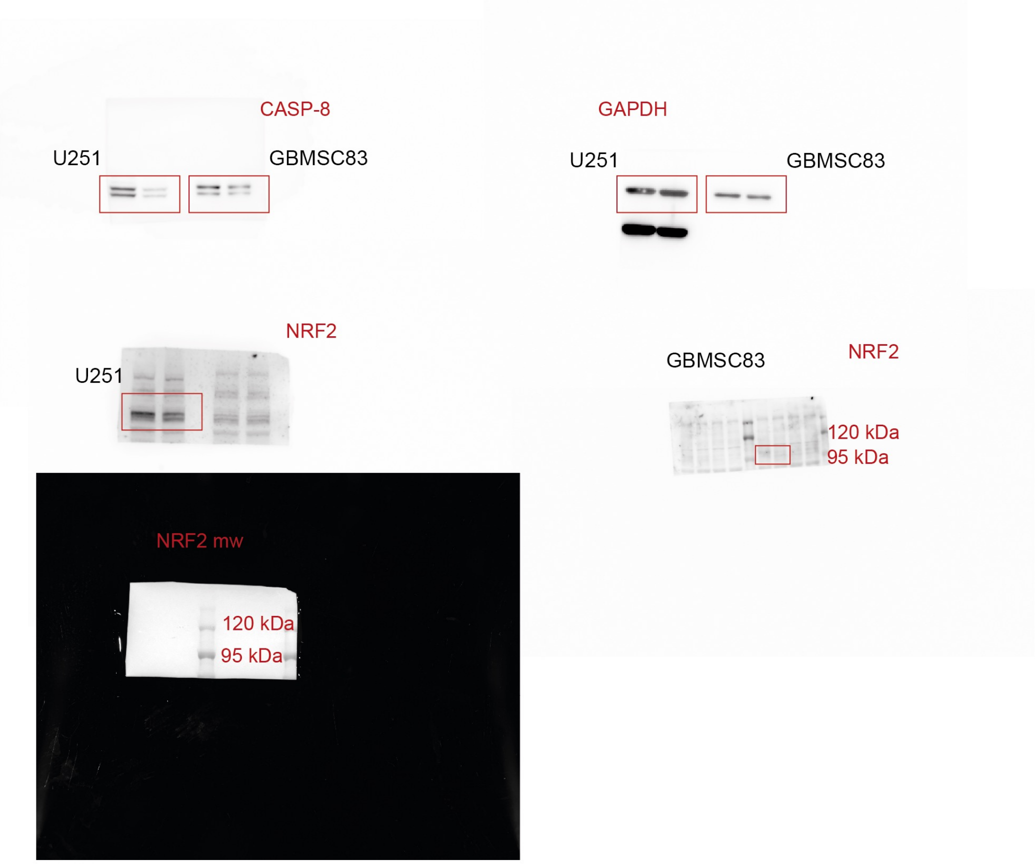

Original WB - Figure 3

Figure 3C

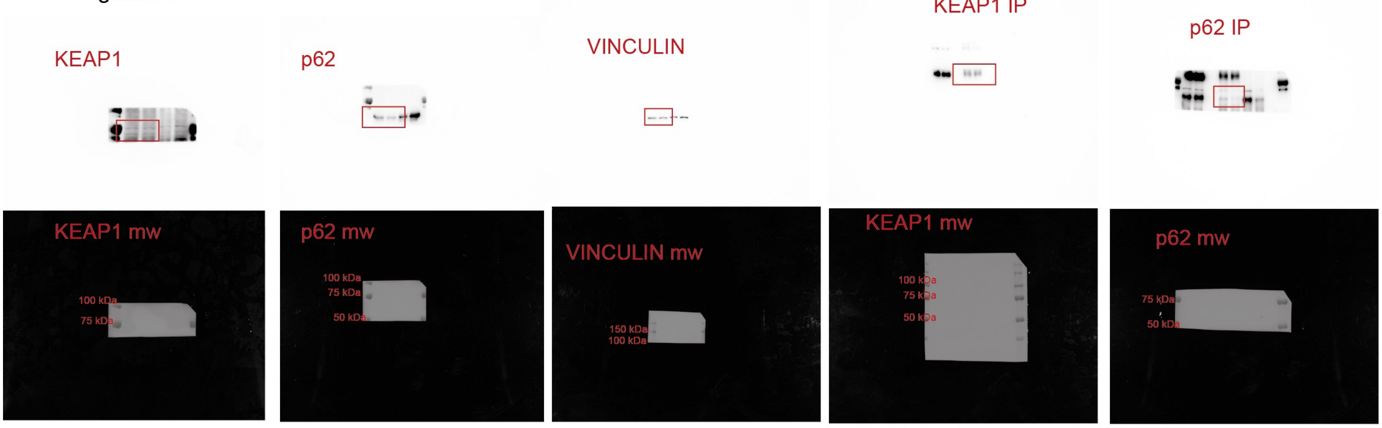

Figure 3D

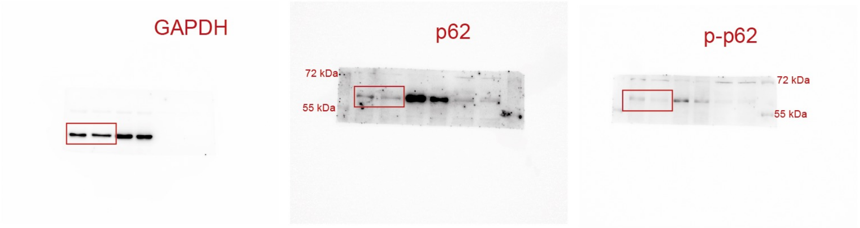

Figure 3E

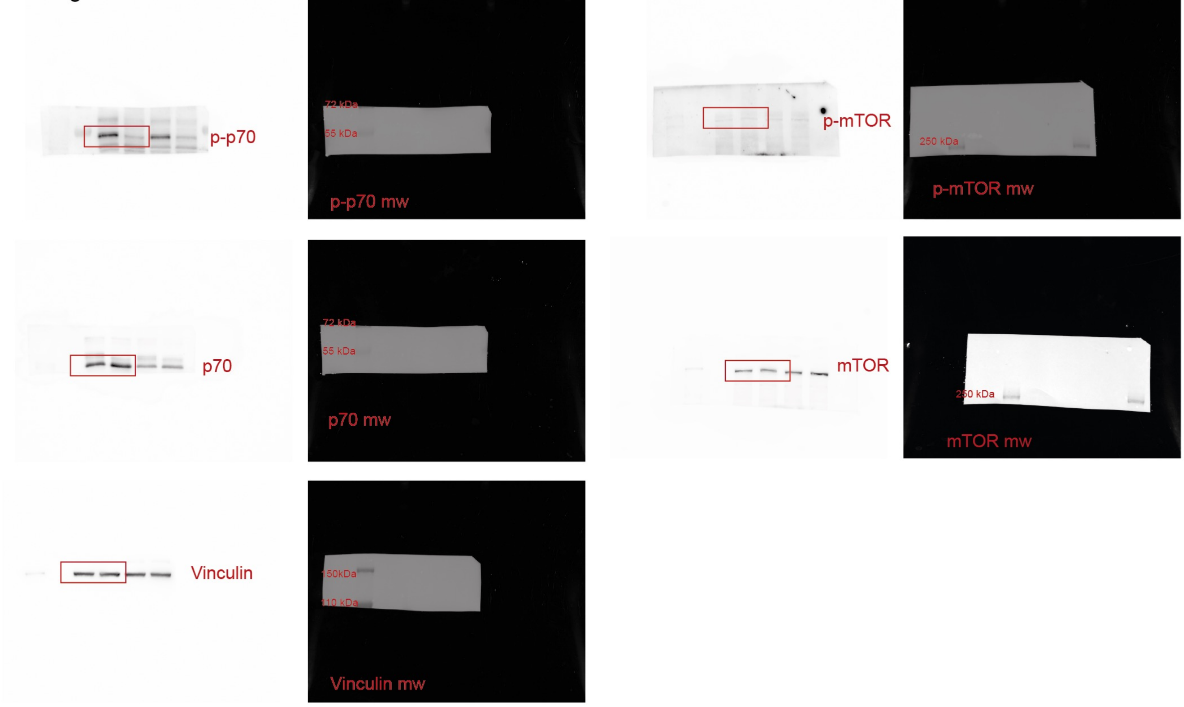

Figure 3G

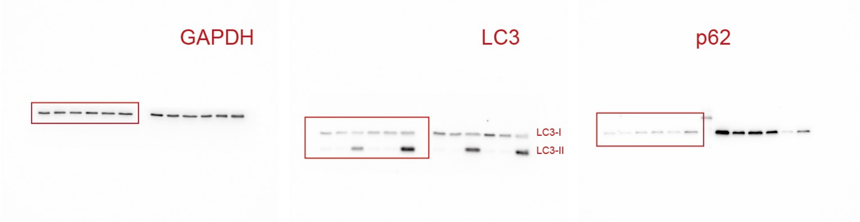

Original WB - Figure 4

4C + S4E

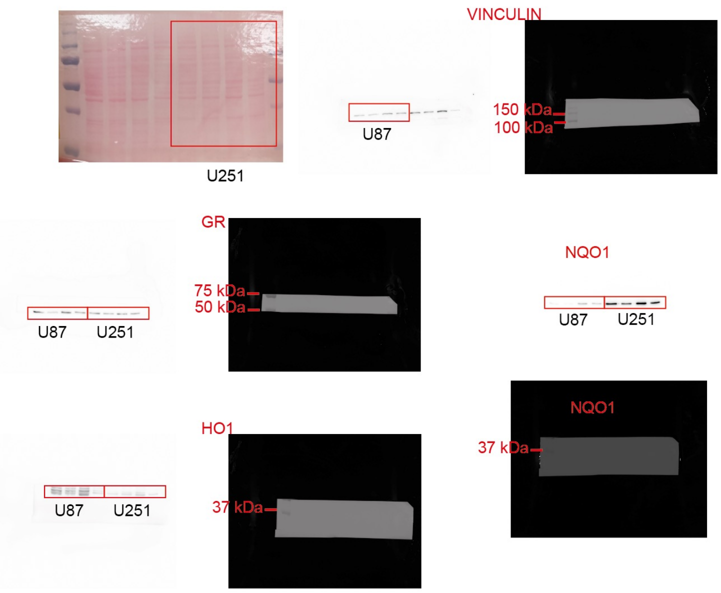

4D

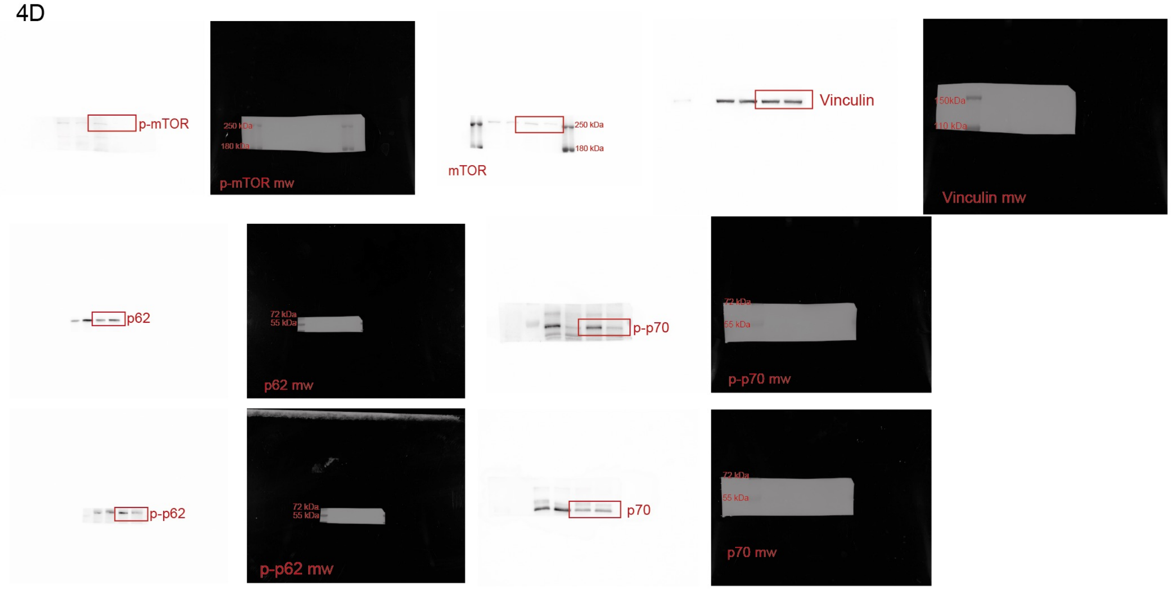

4E

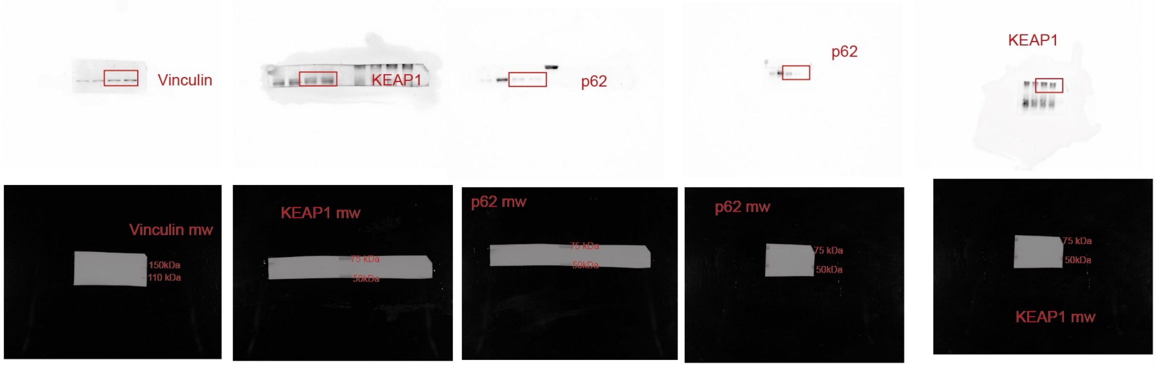

Original WB – Figures 5-6

5 D

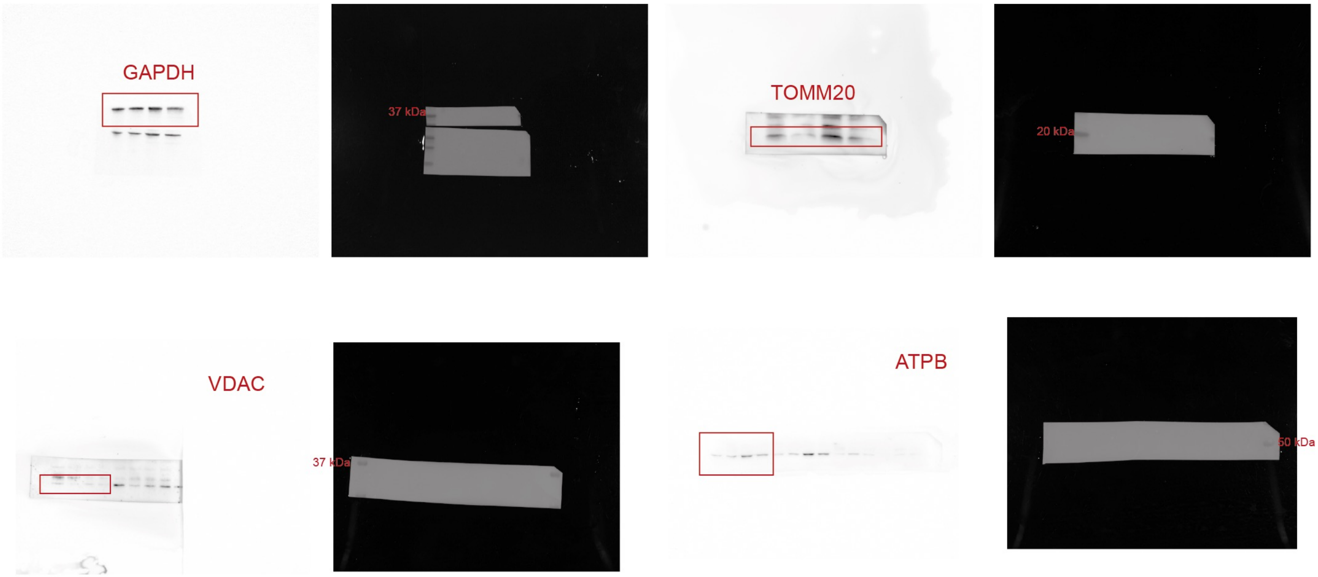

Figure 6

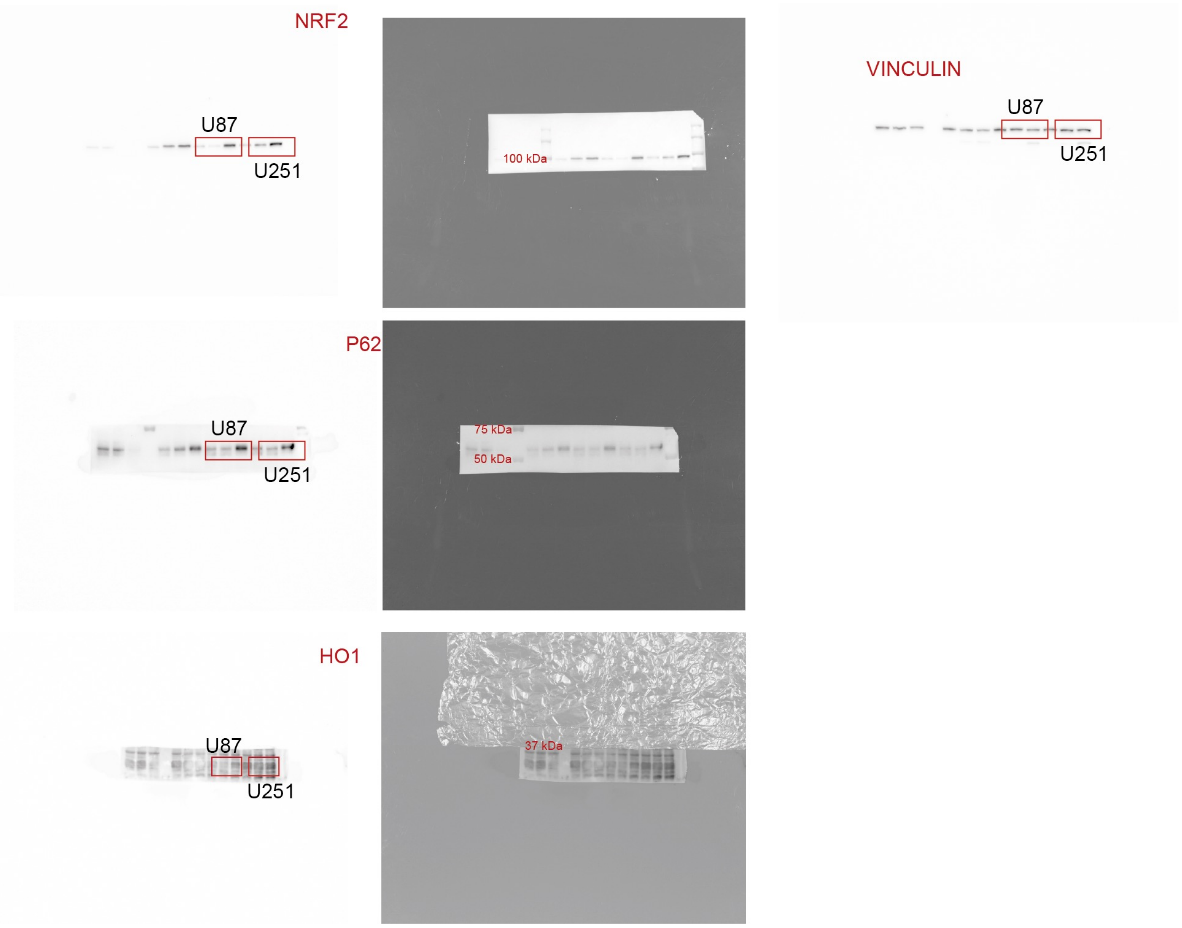

Original WB - Figure S1

S1D

VINCULIN

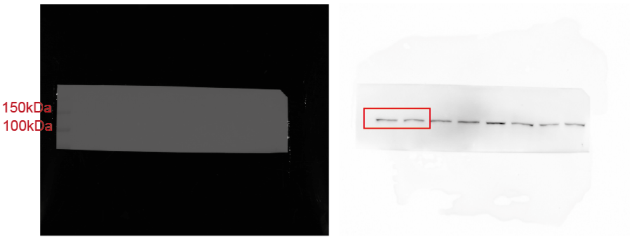

NRF2

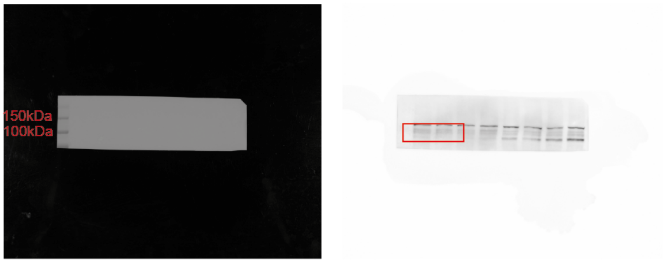

CASP-8

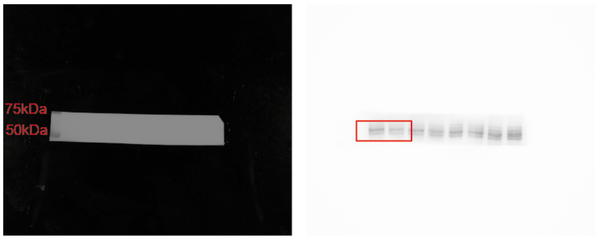

HO1

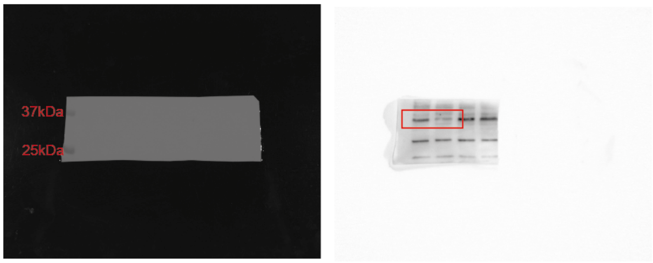

S1G

LAMIN A/C

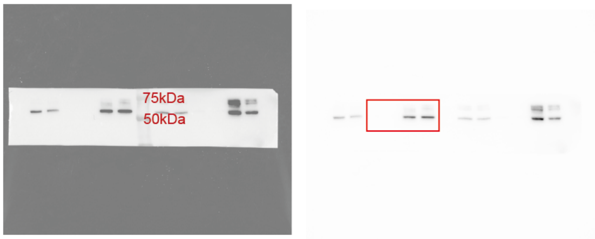

NRF2

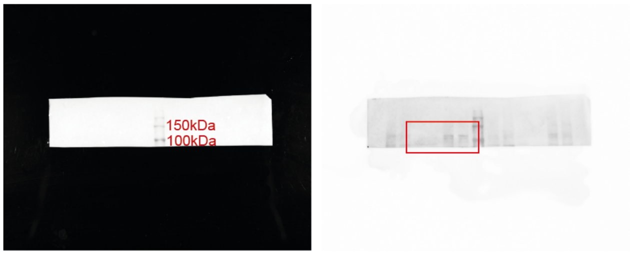

VINCULIN

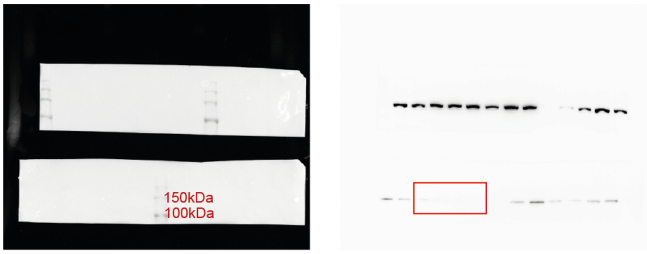

Original WB - Figure S3

S3C

GAPDH

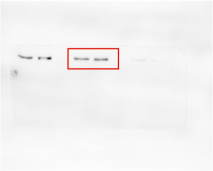

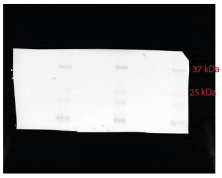

p62

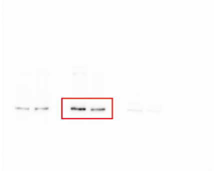

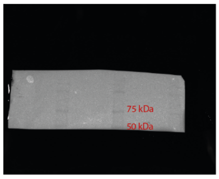

p-p62S349

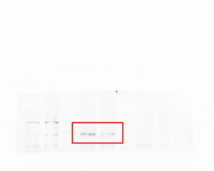

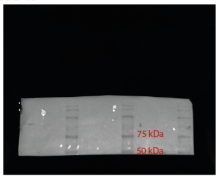

S3D

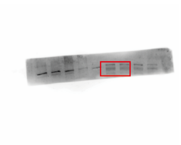

mTOR

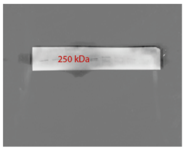

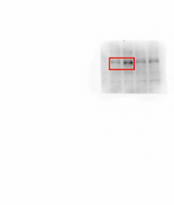

p70 TOT

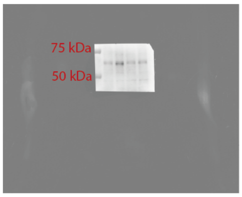

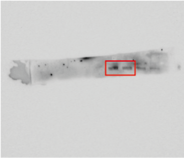

p-mTOR

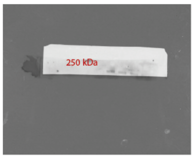

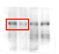

p-p70

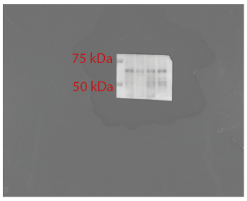

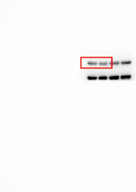

GAPDH

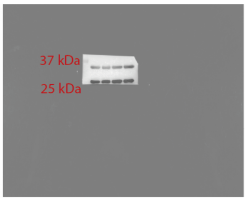

S3P

ACTIN

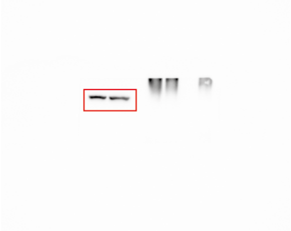

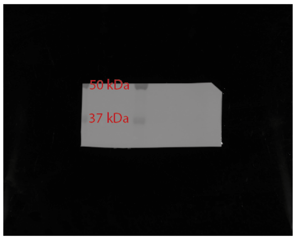

TFEB

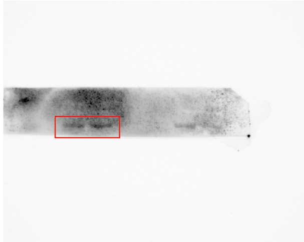

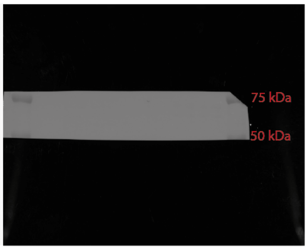

VINCULIN

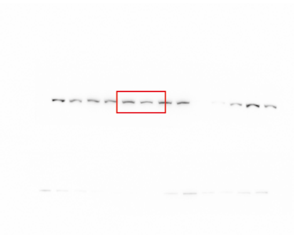

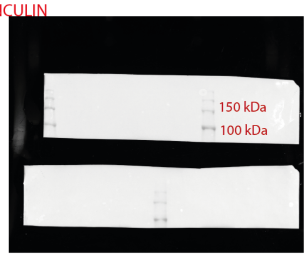

TFEB

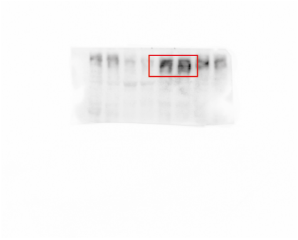

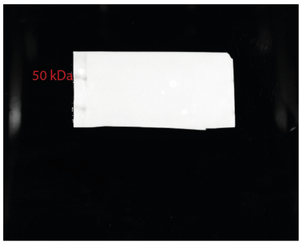

Original WB - Figure S4

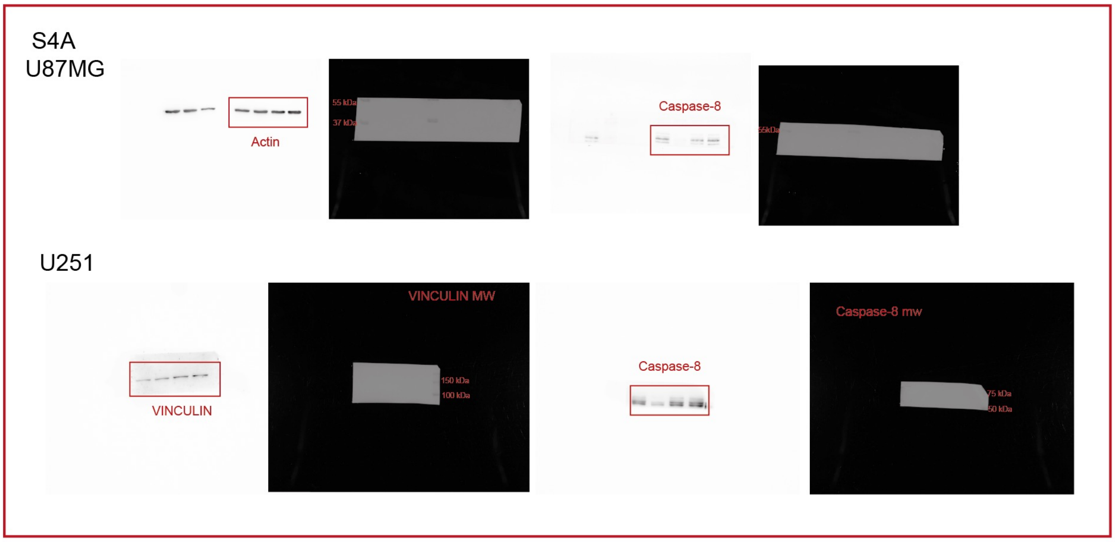

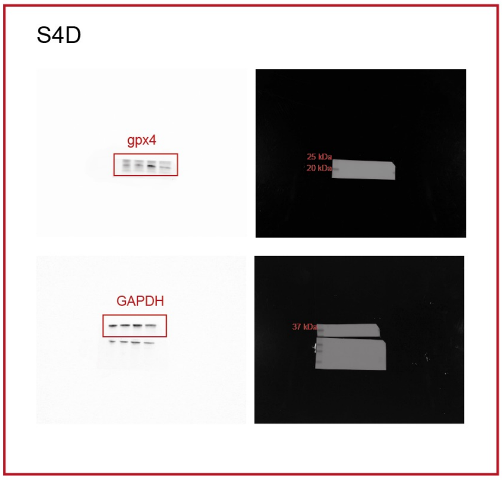

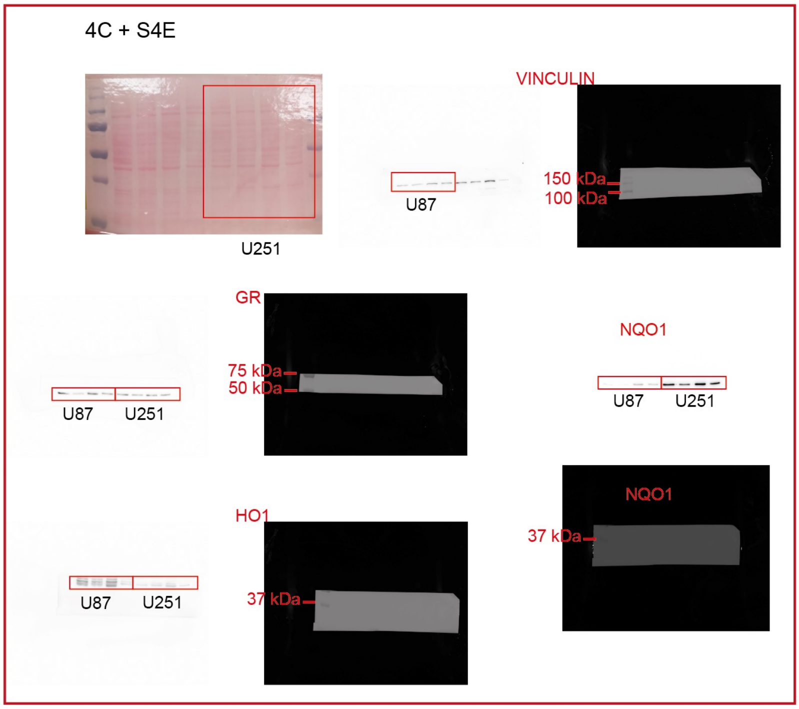

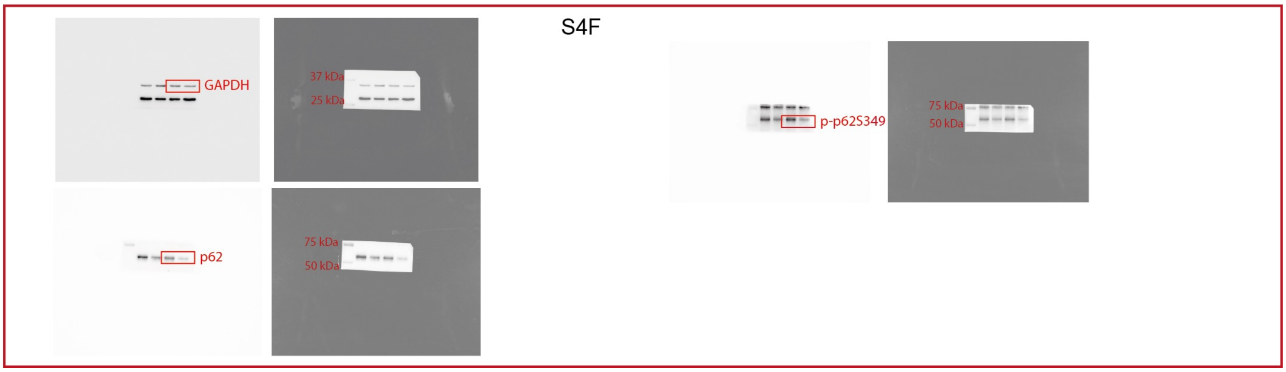

Supplement: Supplementary file 2 — Original Wester Blots [file 41418_2025_1542_MOESM2_ESM.pdf]
